# Conformational Heterogeneity and Interchain Percolation Revealed in an Amorphous Conjugated Polymer

**DOI:** 10.1021/acsnano.2c04794

**Published:** 2022-09-14

**Authors:** Robert M. Ziolek, Alejandro Santana-Bonilla, Raquel López-Ríos de Castro, Reimer Kühn, Mark Green, Christian D. Lorenz

**Affiliations:** † Biological Physics and Soft Matter Group, Department of Physics, King’s College London, London WC2R 2LS, United Kingdom; ‡ Department of Physics, King’s College London, London WC2R 2LS, United Kingdom; ⊥ Department of Chemistry, King’s College London, London, SE1 1DB, United Kingdom; § Department of Mathematics, King’s College London, London WC2R 2LS, United Kingdom; ∥ Photonics and Nanotechnology Group, Department of Physics, King’s College London, London WC2R 2LS, United Kingdom

**Keywords:** molecular dynamics simulations, density functional theory, conjugated polymers, machine learning, graph theory, percolation, molecular conformation

## Abstract

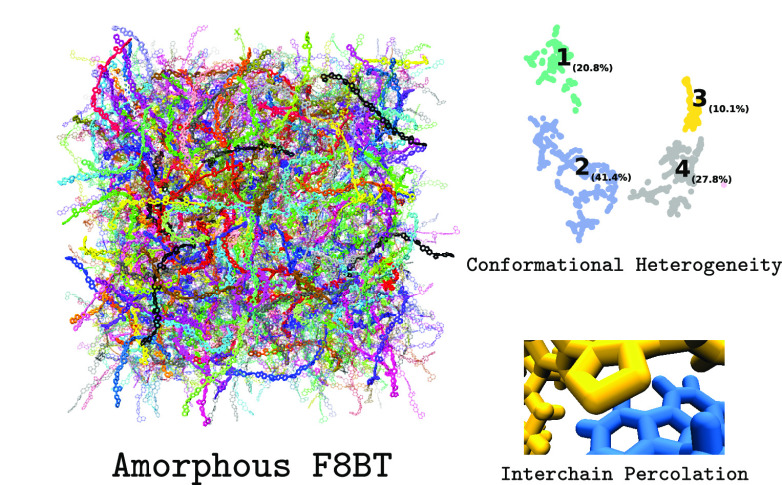

Conjugated polymers are employed in a variety of application areas due to their bright fluorescence and strong biocompatibility. However, understanding the structure of amorphous conjugated polymers on the nanoscale is extremely challenging compared to their related crystalline phases. Using a bespoke classical force field, we study amorphous poly(9,9-di-*n*-octylfluorene-*alt*-benzothiadiazole) (F8BT) with molecular dynamics simulations to investigate the role that its nanoscale structure plays in controlling its emergent (and all-important) optical properties. Notably, we show that a giant percolating cluster exists within amorphous F8BT, which has ramifications in understanding the nature of interchain species that drive the quantum yield reduction and bathochromic shift observed in conjugated polymer-based devices and nanostructures. We also show that distinct conformations can be unravelled from within the disordered structure of amorphous F8BT using a two-stage machine learning protocol, highlighting a link between molecular conformation and ring stacking propensity. This work provides predictive understanding by which to enhance the optical properties of next-generation conjugated polymer-based devices and materials by rational, simulation-led design principles.

The intrinsic fluorescence brightness, photostability, and biocompatibility of conjugated polymers (CPs) underlie their adaptation in a broad range of functional applications in medical therapy,^1^ biological imaging,^2^ sensing,^3^ and organic and bioelectronics.^4−7^ For these applications, readily fabricated conjugated polymer nanoparticles (CPNs) are formed using capping agents to provide stable and soluble conjugated polymer formulations.^8^ Postassembly functional modification and chemical doping can be used to further tune CPN properties for target applications.^9,10^

Our understanding of conjugated polymers is built on a broad range of research. The effects of internal dihedral rotation upon the emission spectrum and efficiency of conjugated polymer thin films have been studied experimentally,^11^ while in highly doped conjugated polymers, paracrystalline disorder, rather than the ionic size of the doping ions, has been shown to control charge transport.^12^ Predicting crystal structures of different conjugated polymers and how this structure impacts their properties is a current area of research;^13^ however, a higher degree of crystallinity has been shown to not necessarily lead to an increase in charge-carrier mobility.^14^ Recently, cryogenic tunnelling electron microscopy has been used to study the nanoscale structure of CPNs.^15^ This approach extends capabilities to identify crystalline subregions in CPNs; however, a full characterization of the amorphous regions with similar detail remains elusive.

Accompanying experimental research is an ever-growing collection of computational and theoretical studies, led primarily by density functional theory (DFT) and related methods, which can provide detailed mechanistic understanding into the optical properties of conjugated polymers on the single molecule level. DFT has been applied to examine how the internal rotation of conjugated polymers determines their magnetic-electronic coupling,^16^ to validate the use of more computationally efficient semiempirical methods to virtually screen conjugated polymers,^17^ and to provide computational predictions of the changing electronic and optical properties of conjugated polymers in response to charge injection,^18^ to list just a few among many examples.^19,20^ However, DFT calculations are typically limited in application to relatively small system sizes given their computational cost and cannot be used to simulate the long time and large length scales required to study amorphous conjugated polymers.

The nanoscale structure of conjugated polymer materials emerges from interactions between different molecules and holds significant control over their much-prized optical properties.^21−24^ Reliably controlling the optical properties of conjugated polymers upon aggregation and assembly remains a challenge.^25,26^ While the intrinsic periodicity of crystalline phases of conjugated polymers makes them more readily amenable to study by different theoretical and computational methods,^27^ understanding the structure of amorphous conjugated polymers is more challenging. Classical molecular dynamics (MD) simulations are typically the computational method of choice to investigate polymer-based materials at the nanoscale, offering the prospect of understanding the behavior of individual molecules within amorphous conjugated polymers in great detail. There are comparatively few examples of all-atom MD simulations of conjugated polymers^28−30^ compared to other classes of polymers, principally due to the difficulty and computational cost of developing suitable force field parametrization schemes to accurately model them.^29^

In this work, we study poly(9,9-di-*n*-octylfluorene-*alt*-benzothiadiazole) (F8BT, whose chemical structure is shown in Figure S1), an alternating donor–acceptor conjugated polymer whose strong fluorescent brightness, high quantum yield, and large ionization potential have made it a model conjugated polymer, deployed in diverse application areas.^31,32^ While it has been extensively studied experimentally, corresponding computer simulation studies of F8BT have not yet been reported, and a clear understanding of its amorphous state remains lacking. Using a bespoke classical force field that we reparameterized using DFT, we simulated a large amorphous F8BT system and F8BT chains in both tetrahydrofuran (THF) and water. As well as revealing the structure of an amorphous conjugated polymer in detail, our results directly demonstrate proposed origins of the reduced quantum yield of CPNs relative to solvated conjugated polymer chains^33^ and provide a framework to understand the bathochromic shift in CP-based devices from an atomistic perspective. In doing so, we highlight the value of classical MD simulations in understanding the emergent properties of amorphous conjugated polymers that are currently inaccessible by quantum chemical methods, even when applying state-of-the-art computational resources.

## Results and Discussion

### Structural Properties of Individual F8BT Chains in Solution and in the Amorphous Phase

**Figure 1:**
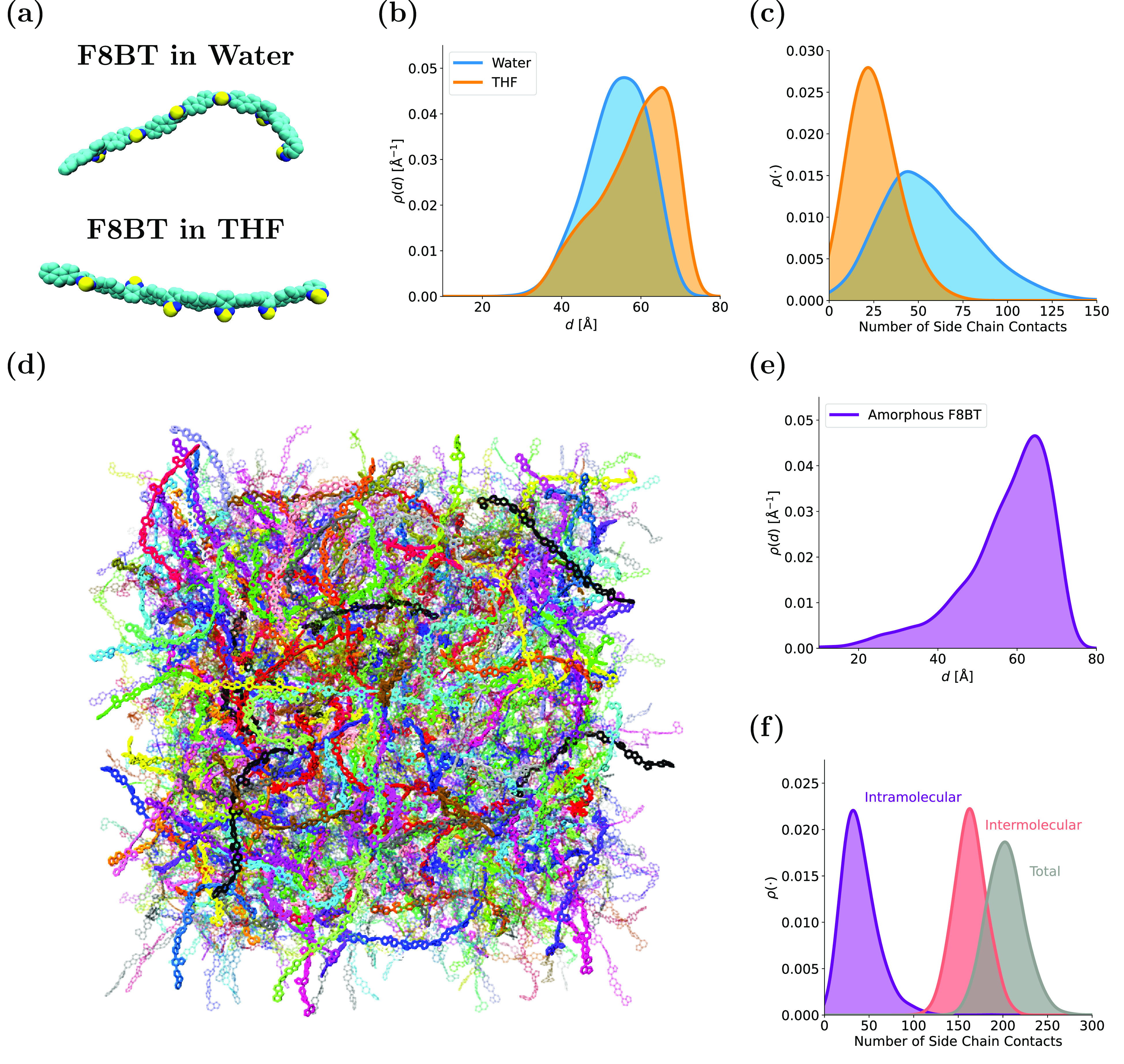
Structural properties of individual F8BT chains in solution and in the amorphous phase. (a) Snapshots of F8BT chains in solution at their most probable extension length in water and THF (side chains not shown). (b) Distributions of F8BT end-to-end distances in water and THF. (c) Distributions of the number of F8BT side chain contacts in water and THF. (d) Snapshot of amorphous F8BT with individual chains shown in different colors. (e) Distribution of chain end-to-end distances in amorphous F8BT. (f) Distribution of the number of side chain contacts in amorphous F8BT.

Figure 1a shows snapshots of F8BT chains in water and THF at their respective most probable end-to-end lengths. As expected, the F8BT chain backbone in THF is more extended than in water. This is reflected in the corresponding probability density distributions of the chain end-to-end lengths (*d*) (Figure 1b), with THF conferring a greater mean degree of extension (*d̅* = 58.2 ± 8.9 Å) than water (*d̅* = 54.2 ± 7.8 Å). While the *p*(*d*) distribution for F8BT in water is reasonably symmetric (the most probable value of *d*, *d̂* ≃ 56 Å ≈ *d̅*), while the distribution in THF is negatively skewed (*d̂* ≃ 65 Å > *d̅*). This highlights that although highly extended chain conformations are most probable in THF, there is also a population of more tightly folded chains. These differences in chain backbone extension, imparted by interactions with surrounding solvent molecules, can be rationalized by determining the number of side chain contacts (Figure 1c). Interactions between the octyl side chains drive the hydrophobic collapse of the F8BT molecule in aqueous solution, as the hydrophobic alkyl chains interact with each other to reduce interactions with the surrounding water molecules. The average number of side chain contacts for F8BT in water (56 ± 26) is significantly greater than for the chain in THF (25 ± 13). The extension lengths (*l*) of the side chains reflect this: They are more contracted in water (*l̅* = 8.2 ± 1.3 Å) than in THF (*l̅* = 8.9 ± 0.9 Å), which implies that the octyl side chains contract toward the polymer backbone to mutually interact and shield themselves from water due to the hydrophobic effect. The solvation of F8BT in water and THF is discussed in detail in the Supporting Information (‘Solvation Mechanisms of F8BT in Water and THF’).

At the high temperature (800 K) initially used in the simulations of amorphous F8BT, individual chains diffuse throughout the simulation box and their conformations evolve significantly over time. After thermally quenching the system, the final temperature of the amorphous F8BT production simulations (350 K) is below the glass transition temperature observed to occur at *T*_g_ ≃ 478 K. This value is significantly higher than the *T*_g_ = 398 K reported experimentally.^34^ Discrepancies between *T*_g_ values calculated by experiment and computer simulation are commonplace,^35^ with differences of the magnitude observed in this work attributable primarily to force field effects,^36^ while the thermal quenching rate also makes a contribution.^37,38^ At *T* < *T*_g_, each F8BT chain is kinetically trapped in a single conformation in the metastable (nonequilibrium) amorphous phase, unlike the single chains studied in solution. As such, we ran two replica simulations to yield suitable statistics and to assess the consistency of our system preparation methodology. We observed no significant differences between the replicas, and as a result, data from both of them are combined for our analysis purposes throughout this work.

A visualization of amorphous F8BT after quenching is presented in Figure 1d, with individual molecules highlighted in different colors. The disorder within the amorphous F8BT is clearly observable, with chains taking on a wide variety of different conformations. This is reflected by the probability density distributions of chain extension lengths in Figure 1e. The mean chain extension length (*d̅* = 57.3 ± 11.1 Å) and most probable chain extension length (*d̂* ≃ 65 Å > *d̅*) are both comparable to the corresponding results for THF. While the summary statistics of the amorphous F8BT and THF distributions are comparable, there is a clear qualitative difference between the two distributions: A heavy tail is present for *d* < 30 Å in amorphous F8BT, unlike for the F8BT chain in THF. We infer that F8BT chains in the amorphous phase take on a wider variety of conformations than their counterpart chain solvated in THF. Furthermore, it is observed that there exists a population of more tightly folded conformations in amorphous F8BT than is present in water. The average number of intramolecular side chain contacts (39 ± 19, which lies between the corresponding results for F8BT in water and THF) is much lower than the average number of intermolecular contacts (167 ± 16, total combined value of side chain contacts is 206 ± 20, see Figure 1f). The average side chain extension length in amorphous F8BT (*l̅* = 8.7 ± 0.9 Å) is also found to lie between the corresponding values for water and THF.

### Interchain Species Percolation in Amorphous F8BT

**Figure 2:**
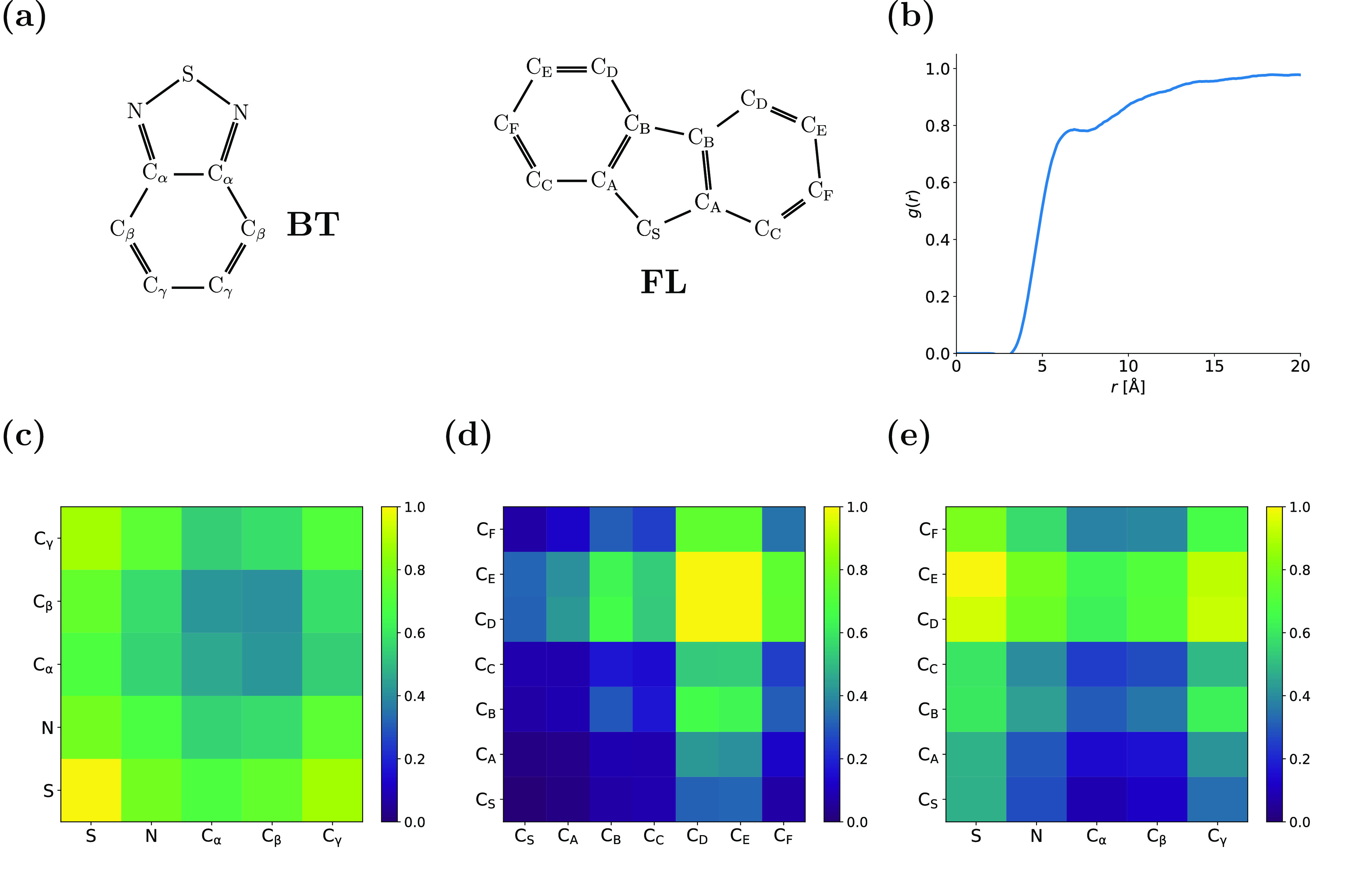
Interactions that drive ring stacking in amorphous F8BT. (a) Nomenclature used for the contact maps to describe specific atomistic interactions between different rings. (b) Ring center of geometry pair distribution function. Contact maps for the different rings of F8BT: (c) BT-BT, (d) FL-FL, and (e) BT-FL. Note that the scale bar for each of the contact maps indicates the relative contact propensity (i.e., a value of 1 corresponds to the most probable interaction). Each contact map is independently normalized.

Ring stacking interactions within conjugated polymers, including F8BT, are of particular interest since these interactions play a vital role in controlling the optical properties of conjugated polymer-based materials.^21^ The effect of ring stacking upon the emission properties of conjugated polymers has been widely studied experimentally, but a detailed network-level picture of this phenomenon derived from classical MD simulations has not previously been realized. Chemical structures of both the benzothiadiazole (BT) and fluorene (FL) moieties and the specific atom labeling nomenclature adopted here are shown in Figure 2a. Figure 2b shows the ring stacking pair distribution function (i.e., the pair distribution function of ring centers of geometry). A small peak is centered at *r* ≈ 6 Å, whose presence implies attractive interactions between rings, which manifest through ring stacking. The contact maps in Figure 2 highlight the atomistic interactions that drive ring stacking within amorphous F8BT. The most probable BT-BT interactions occur between sulfur atoms and C_γ_ (Figure 2c), which is driven by the dipole over the BT ring. A depletion of charge at C_γ_ and a concentration of charge at S means that the dipole stacking mechanism observed experimentally in F8BT is reproduced by our classical force field.^11^ While atoms in the BT rings interact relatively universally with those in other BT rings, there is a qualitative difference in the FL-FL (Figure 2d) contact map. While C_D_ and C_E_ interact strongly with each other, steric hindrance imparted by the octyl chains (connected at C_S_) hinder interactions with and between the other atoms in the FL ring. This implies that FL-FL stacking may be more staggered than BT-BT stacking. The FL-BT contact map (Figure 2e) shows evidence of similar steric effects from the octyl side chains: The most probable interactions arise at the C_D_ and C_E_ atoms of FL rings once more.

**Figure 3:**
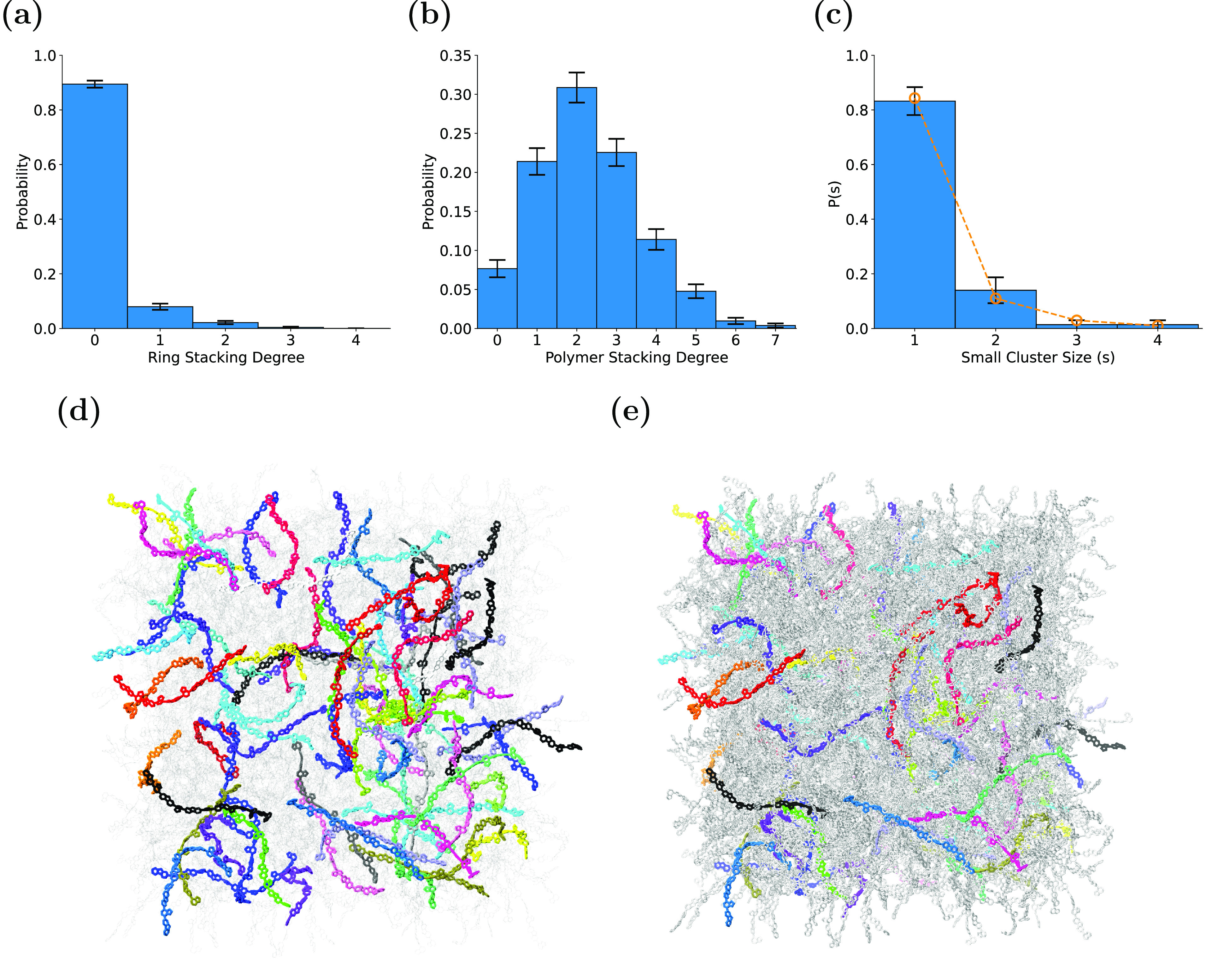
A GPC emerges from ring stacking in amorphous F8BT. (a) Individual ring stacking degree and (b) overall degree of stacking per polymer. (c) Size distribution of small clusters in amorphous F8BT, with results from theoretical random graph model shown in orange. Note that all error bars show the 90% CI. Visualizations from a single replica showing (d) all polymers found in small clusters in different colors (polymers in the GPC are shown as translucent) and (e) same as (d) but with polymers in GPC shown in gray.

To understand the nature of ring stacking throughout amorphous F8BT, we use graph theory to analyze and model the emergent properties of the ring stacking network. See the Network Analysis of Ring Stacking in Amorphous F8BT section for details regarding the construction of the different graphs we consider here. The ring stacking propensity within amorphous F8BT is first examined by calculating the degree of stacking per ring (i.e., the number of stacking interactions a given ring undertakes). Figure 3a shows the probability distribution of the ring stacking degree, with the average degree of stacking shown to be low (≈ 0.14) with only ≈11% of the individual rings in amorphous F8BT interacting with another ring by stacking. There are a small number of rings that are involved in more than one ring stacking interaction, which can be a result of a given ring either stacking with other rings both above and below itself or by staggered stacking interactions occurring between multiple rings. Note that all of the error bars in Figure 3 represent the 90% confidence interval (CI = *x̅* ± 1.645σ/√*n*, where *x̅* is the sample mean, σ the standard deviation, and *n* the number of data).

A rather different picture emerges when considering the stacking degree per polymer, as demonstrated by the probability distribution in Figure 3b. The vast majority of polymers (approximately 92%) are observed to be undertaking at least one stacking interaction, with an average of 2.3 ± 1.3 stacking interactions per polymer. By analyzing the cluster size distribution of , we observe that a giant percolating cluster (GPC) is formed in both replica simulations, containing 89% of F8BT molecules within the system (one would not expect significant finite size effects to affect our simulation result so long as we remain far from the percolation threshold). Interestingly, the simulation-derived result is in excellent agreement with the corresponding theoretical result for an infinite random graph model initialized with the same degree distribution: A GPC containing 88.78% of all polymers is identified. This suggests not only that our simulations reproduce the same GPC size as our theoretical results but also that the infinite random graph model may be of use in modeling charge transport phenomena in conjugated polymers more broadly. As well as the percolating cluster, a variety of small clusters exist within amorphous F8BT. Figure 3c shows the distribution of small (nonpercolating) clusters from the simulations and from the infinite random graph model (orange circles), for which full results are shown in Figure S12. Again, these results are in excellent mutual agreement, highlighting that the (mean field) infinite random graph is a good model for the simulation results. This information may prove useful as a measure to understand how large simulated systems must be in order to effectively mitigate finite size effects on network level properties in the future. Figure 3d shows the F8BT chains not found in the GPC in different colors (i.e., those in small clusters). For comparison, Figure 3e more clearly depicts the F8BT chains that make up the GPC in gray.

Given that the formation of interchain species is responsible for the bathochromic (red) shift in the CPN emission band and the reduction of quantum yield of conjugated polymer-based devices compared to the same CP in solution,^21^ understanding the network-level properties of F8BT in the simulations is of great interest. This reduction in quantum yield reduces the effectiveness of CPNs as imaging agents and in their other various applications. The change in emission wavelength is also problematic in the production of CP-based devices. The experimentally observed bathochromic shift in conjugated polymers is attributed to the delocalization of electron density over different molecules that make up interchain species, which lowers the interchain species electronic energy relative to the single chain. Subsequently, the low degree of overlap between the delocalized excited state and the single chain electronic ground state wave function gives rise to long radiative lifetimes in CP-based materials^39^ and with it the enhancement of nonradiative relaxation pathways and subsequent reduction in quantum yield as compared to single CP chains in solution.^40^ Our results highlight that even in nonannealed, amorphous F8BT, the relatively sparse ring stacking interactions present in the system facilitate the emergence of a GPC. With annealing, one would expect the degree of ring stacking to increase and with it a larger, and more strongly connected, GPC to emerge. We infer that a high level of stacking interactions between molecules, in excess of those required to yield a GPC, are required to facilitate effective charge delocalization in conjugated polymer-based materials and drive significant reductions in their quantum yield.

### Unsupervised Learning Reveals Distinct Conformational Clusters in Amorphous F8BT

In addition to ring stacking, the conformations adopted by individual conjugated polymer chains within thin films and nanoparticles also affect their all-important emission properties.^21^ In the case of semiconductor quantum dots, obtaining detailed structural information on their underlying lattice structure is possible using various high-energy methods.^41^ These techniques are not suitable to probe the structure of CPNs; however, cryogenic tunneling electron microscopy has been recently used to study the structure of CPNs, showing that ordered crystalline regions exist within otherwise a broadly amorphous environment.^15^ The identity of the crystalline regions identified can then be resolved by comparing their lattice spacing to results from polymer thin films. However, the structure of the amorphous regions still cannot be further resolved experimentally. We now show how analyzing MD simulations of amorphous F8BT with a two-stage machine learning approach can be used to determine the conformational landscape of F8BT in the amorphous phase in great detail.

**Figure 4:**
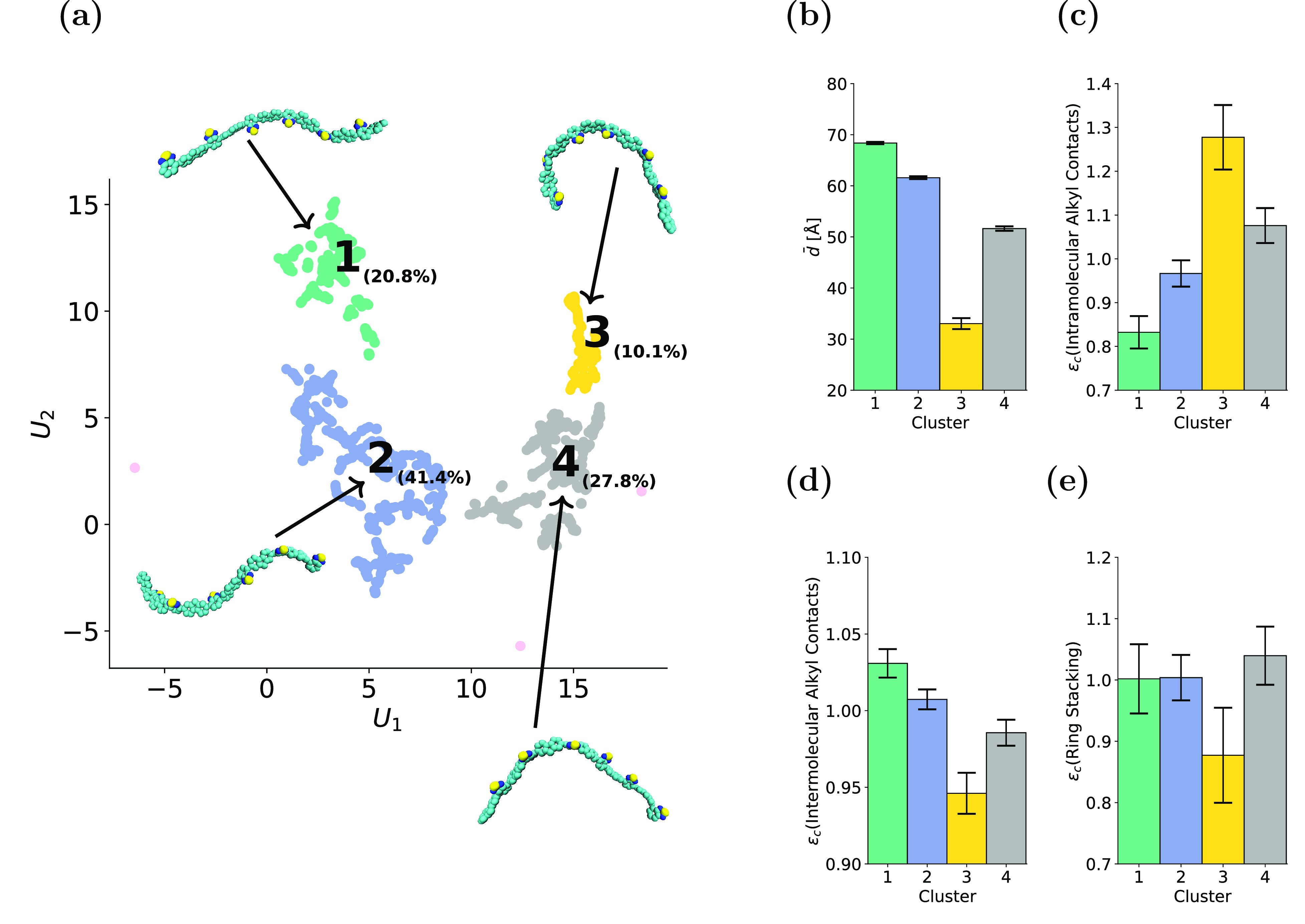
Unsupervised learning reveals distinct conformational clusters in amorphous F8BT. (a) UMAP embedding of the conformational distribution with amorphous F8BT. A representative snapshot is shown for each cluster. (b) Average end-to-end backbone distance (*d̅*) of each cluster. (c) Enrichment of intramolecular alkyl chain contacts, (d) enrichment of intermolecular alkyl chain contacts, and (e) enrichment of ring stacking. Note that the error bars show the 90% CI.

To briefly recap, we first use the UMAP algorithm to perform dimensionality reduction from a high-dimensional input space (a set of through-space distances that seek to describe the overall conformation of each polymer) to a two-dimensional embedding. As well as making direct visualization of the original data possible, this procedure also allows us to subsequently perform density-based clustering in the two-dimensional embedded space, pragmatically mitigating against the curse of dimensionality. The embedded space representation of the conformational distribution in amorphous F8BT (using data from both replica simulations on an equal footing) is shown in Figure 4a. Details of the exact procedure and hyperparameter assignment are provided in the Model and Methods section. Four conformational clusters were identified using HDBSCAN; their relative abundance is indicated as a percentage in Figure 4a, and snapshots of representative molecules from each cluster are shown. The average end-to-end extension lengths for each cluster are shown in Figure 4b. Immediately clear is the variety of structures identified: The snapshots for clusters 1 and 2 depict generally extended conformations, while those for clusters 3 and 4 are folded conformations. Cluster 1 is the most extended (*d̅* = 68.3 ± 0.2 Å), while cluster 3 is the most folded (*d̅* = 33.0 ± 1.1 Å). Kinetically trapped, highly folded conformations account for 10.1% of the total (see Figure 4a). These results show that, in our system at least, there is not simply a single collapsed state in amorphous F8BT but rather a distribution of distinct states with different amounts of folding. These different conformational states underlie the broad distribution of end-to-end distances exhibited by amorphous F8BT in Figure 1e, with the tightly folded conformational state, identified here as cluster 3, accounting for the heavy tail at low *d* values.

There is an inverse relationship between *d̅* (Figure 4b) and the average number of intramolecular alkyl chain contacts for the different clusters (Figure 4c). For the most folded conformations (cluster 3), there is a significant enrichment of intramolecular alkyl chain contacts. The relaxation of octyl side chains can be quantified by considering the long time limit of the side chain root-mean-squared-deviation, Γ_max_, as described in the Supporting Information (‘Quantifying Polymer Deformation and Relaxation’).^42^ For octyl side chains in THF, Γ_max_ = 6.9 Å, which is indicative of side chains moving freely in solution. For the octyl side chains in amorphous F8BT after cooling, a much lower value of Γ_max_ = 2.0 Å is calculated, which corresponds to highly restricted local fluctuations of the side chains over time. Since there is no large-scale conformational evolution of the conjugated polymer backbone or side chains at *T* < *T*_g_, we infer that tightly folded polymer conformations (e.g., cluster 3 in Figure 4) exist at high temperatures, with their side chains undertaking significantly more intramolecular interactions than the more extended conformations within amorphous F8BT. The opposite relationship between *d̅* and the average number of intermolecular alkyl chain contacts is observed (Figure 4d); in more extended molecules, side chains are more likely to interact with those of other molecules. The absolute differences in the distributions shown in Figure 4c,d are a result of the relative magnitudes of the two quantities given the enrichment definition. Figure 4e shows the stacking enrichment of the different clusters, which is suppressed in cluster 3. This result directly demonstrates that ring stacking is hindered in highly folded F8BT conformations and, as such, provides evidence that folded polymer conformations are intrinsically linked to reduced ring stacking propensity in amorphous F8BT.

The use of cyclodextrins^43^ and side chain modification^44^ as insulating species^45^ to improve the emission efficiency of CPs has been reported, while the exact mechanism of their action remains elusive. The highly folded states observed in amorphous F8BT in this work can be considered to be acting as insulators to some degree since they do not interact as frequently via ring stacking as do the more extended conformations, which hinders the formation of the giant cluster. This mechanism may play a significant role in controlling the quantum yield of nonannealed CP thin films, which is higher than their annealed counterparts.^46^ Such folded conformations would be removed upon annealing. The presence of small (nonpercolating) clusters identified in the previous section, in addition to the number of distinct conformational clusters identified here, may go some way to explain the slight emission band broadening that has previously been observed for CPNs relative to the same CP in solution,^47^ since one would expect them to each have diverse optical properties.

## Conclusions

Using a bespoke classical force field, we studied the donor–acceptor conjugated polymer F8BT using classical MD simulations. Our results highlight that in amorphous F8BT, a relatively low degree of ring stacking is still able to drive the formation of a large percolating cluster, with ramifications for understanding the reduction in quantum yield and bathochromic shift in the emission spectra of CPNs relative to the same CP in solution. Furthermore, we note a variety of small clusters exist alongside the giant cluster, which is in excellent agreement with theoretical results from an infinite random graph model. The excellent agreement with the theoretical results indicates that the random graph approach may be a useful general framework to study the network level structure of conjugated polymers more broadly. The size of the percolating cluster as identified in this work, which could be controlled by chemical modification of CP side chains or by the addition of insulating species, may be a useful network-level molecular descriptor by which to drive the design of next-generation CPNs with enhanced optical properties. Our two-stage unsupervised learning protocol shows that distinct conformational states exist in amorphous F8BT. A link between molecular conformation and ring stacking efficiency was established, whereby the most folded conformational cluster exhibits ring stacking depletion. Bringing together the conformational and ring stacking information from these simulations with a charge mobility representation within a graph theoretic approach would make for an interesting future research direction.^48^ The detailed picture of amorphous F8BT established in this work provides insight into the nanoscale structure of conjugated polymers in the amorphous state and suggests that MD simulations may become a useful source of design inspiration for next-generation CPNs.

## Model and Methods

### F8BT Force Field Parameterization

All DFT calculations were performed using Orca 4.2.1,^49,50^ using the B3LYP exchange–correlation functional^51−53^ and the Karlsruhe def2-tzvpp basis set.^54^ The VeryTightSCF flag was selected for all calculations (i.e., an energy change tolerance of 1.0 × 10^–9^ au must be met for each geometry optimization to converge). An initial structure of an “F0BT” chain (i.e., an F8BT chain with its octyl side chains replaced by H atoms) was obtained using the semiempirical GFN2-xTB method.^55^ Note that the octyl chains present in F8BT were not included in these calculations since they have been shown to hinder the convergence and accuracy of quantum chemical geometry optimizations.^56^ The optimized geometry of the F0BT chain from DFT was used to calculate the partial charges for the classical force field.^57^ Full details of our procedures and the resultant force field are described in the Supporting Information (‘Reparametrization of the F8BT Force Field’). To test the validity of our F8BT force field, we conducted simulations of multiple F8BT chains in THF and water to observe their aggregation, as assessed by a clustering algorithm.^58^ The agreement between the corresponding results and experimental F8BT solubility in water and THF^59^ highlights that the intermolecular interactions between F8BT atoms are faithfully represented by the force field. Full details of these simulations are provided in Supporting Information (‘Validation of the F8BT Force Field’).

### Molecular Dynamics Simulations

F8BT was modeled using a reparameterized CHARMM-based force field^60,61^ developed as part of this work. Before use in simulations, an F8BT chain was subject to high temperature dynamics in vacuo to randomize its intermonomer dihedral distribution. We note that frequent transitions between different dihedral states are observed in all simulations before statistics are calculated. Water was treated with the CHARMM-modified TIP3P model,^62^ and THF was modeled using the CHARMM36 force field.^60,61^ All of the MD simulations reported in this work were performed using the GROMACS 2019 simulation engine.^63,64^ In all simulations, the Lennard-Jones and Coulomb interaction cutoff distances were set to 12 Å. The particle-mesh Ewald method was used to calculate long-range electrostatic interactions. Periodic boundary conditions were applied in all dimensions in all simulations. A 1 fs time step was used for all equilibration and production simulations. The leapfrog integration scheme was used in all simulations unless otherwise detailed. Details of the simulated systems are provided in Table 1.

**Table 1:** Details of Each of the Simulated Systems

system	*n*_atoms_	*n*_F8BT_	*n*_solvent_	final box size (Å^3^)
amorphous F8BT^a^	374,032	776	–	158.4 × 158.4 × 158.4
F8BT in H_2_O	98,288	1	32,602	100.8 × 100.8 × 100.8
F8BT in THF	96,773	1	7407	104.3 × 104.3 × 104.3

a Two amorphous F8BT replica simulations were performed.

#### Amorphous F8BT Simulation

To begin building the amorphous F8BT system, 100 F8BT molecules were placed in a 200 × 200 × 200 Å^3^ simulation box. Energy minimization using a steepest descent algorithm was performed to remove any high-energy steric clashes. An initial aggregate of 97 molecules formed in the NVT ensemble using the Berendsen thermostat (temperature kept constant at 800 K).^65^ This aggregate was extracted and replicated eight times to yield the initial F8BT melt system containing 776 F8BT molecules. Once more, energy minimization using a steepest descent algorithm was performed to remove any high-energy steric clashes. The temperature was then equilibrated to 800 K in the NVT ensemble using the Berendsen thermostat for 100 ps. Subsequently, the pressure of the simulation box was equilibrated for 5 ns using the Berendsen thermostat and Berendsen barostat (target pressure of 1 atm). The production simulation was then conducted using the Nosé–Hoover thermostat and the Parrinello–Rahman barostat to sample from the true NPT ensemble.^66,67^ The pressure was kept constant at 1 atm. The simulation began at a temperature of 800 K for 50 ns. Each F8BT molecule was able to diffuse and change its conformation at this high temperature. Following this, a first cooling stage was performed at a constant rate of 10 K ns^–1^ to a temperature of 600 K. Following 50 ns of simulation time, the simulation was again cooled at the constant rate of 10 K ns^–1^ to the final target temperature of 350 K. Once reached, the simulation was run for a further 15 ns. Note that at 350 K, the F8BT melt is clearly below its glass transition temperature and we observed no structural evolution of the polymer chains. Therefore, only a short simulation at the final temperature was required to allow the side chains to evolve, as no further structural information regarding the polymer backbones could be collected by extending the simulation further. The final length of the simulation box is in excess of 6*R*_g_. Two amorphous F8BT replicas were simulated to collect sufficient data for the subsequent analysis.

#### Simulations of Single Solvated F8BT Chains

For both water and THF simulations, a single F8BT chain was placed in a simulation box measuring 100 × 100 × 100 Å^3^. Following energy minimization of the single chain by steepest descent, solvent molecules were added to fill the box at an appropriate density (see Table 1 for details). The systems were once more subjected to energy minimization by steepest descent before the temperature was equilibrated to 353 K for 100 ps using the Berendsen thermostat. Subsequently, the production simulation for the F8BT chain in water was performed using the Nosé–Hoover thermostat (target temperature of 353 K) and the Parrinello–Rahman barostat (target pressure of 1 atm) for 200 ns. Prior to its production run, the F8BT chain in THF system was further equilibrated in the NPT ensemble using the Berendsen barostat for 3 ns. The production simulation for the F8BT chain in THF was then performed using the Nosé–Hoover thermostat (target temperature of 353 K) and the MTTK barostat^68^ (target pressure of 1 atm) for 200 ns using the velocity Verlet integration scheme, which equivalently ensures sampling from the true NPT ensemble and ensured initial stability of the simulation system.

### Simulation Analysis Techniques

Simulation analysis was performed using in-house Python scripts, which make wide use of the MDAnalysis^69^ and NetworkX packages.^70^ Simulation visualizations were produced using VMD.^71^

#### Identifying Ring Stacking in Amorphous F8BT

While classical force fields do not explicitly account for aromatic ring stacking interactions, they are commonly observed throughout various classes of molecules in classical simulations, arising indirectly through the parametrization scheme, much like hydrogen bonding. We first generate an undirected graph that represents all bonds between nonhydrogen atoms in the F8BT molecules. From this, the NetworkX cycle_basis algorithm can be used to identify all five- and six-membered rings within the polymers. The center of geometry of each ring is calculated and used to find possible ring stacking events using the MDAnalysis distance_array function (i.e., the center of geometries of both rings must be within 10 Å of each other). Ring stacking events are then confirmed by two further criteria being met: First that any two atoms from either ring are within 4 Å of each other, and second that the angle between the two rings is <20° (we also include vectors for which this angle is >160° to account for the arbitrary directionality of the ring normal vector). To calculate the angle between two rings, we use singular value decomposition of the positions of the atoms in each given ring to find its corresponding normal vector. Then the angle between these two normal vectors can be calculated to establish ring stacking interactions. We note that this procedure allows for staggered ring stacking to be suitably identified.

#### Network Analysis of Ring Stacking in Amorphous F8BT

**Figure 5:**
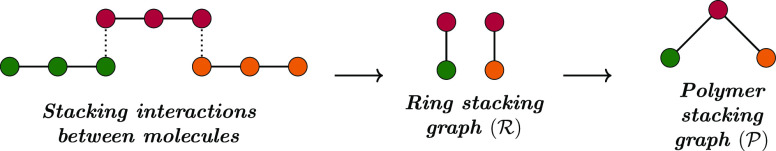
Schematic highlighting the construction of a ring basis graph and a polymer basis graph. Three model polymers are shown in different colors, with solid lines representing covalent bonds between rings (shown as colored nodes) and dotted lines representing intermolecular ring stacking interactions. The two resultant graphs that we use to represent the interactions are shown below (both ring basis and polymer basis graphs).

Figure 5 depicts schematically how the two different graphs we use in this work are constructed. In the ring stacking graph (), nodes represent individual rings in the polymer chains and edges represent the stacking interactions between them. While for the polymer stacking graph (), nodes represent whole polymers and edges represent all the stacking interactions between them (note that this graph remains unweighted). These different network representations allow us to understand ring stacking interactions in different ways. In both cases, we model the network as an unweighted, undirected graph. In Figure 5, the representation of  shows the two pairs of connected rings that emerge in this graph construction (i.e., two clusters of size two), while the representation of  shows that all three polymers are connected in this graph construction (i.e., one cluster of size one). Considering ring stacking as a stochastic process, there are of course multiple chances for stacking interactions to emerge between whole polymers than its individual rings, and as a result, a larger cluster is constructed in  than  from the same constituent ring stacking interactions. Note both graphs are undirected and unweighted. The cluster size distribution that we obtain from  is only weakly influenced by the number of connections each polymer makes to its given neighbors.

#### Unsupervised Learning of Conformational States in Amorphous F8BT

**Figure 6:**
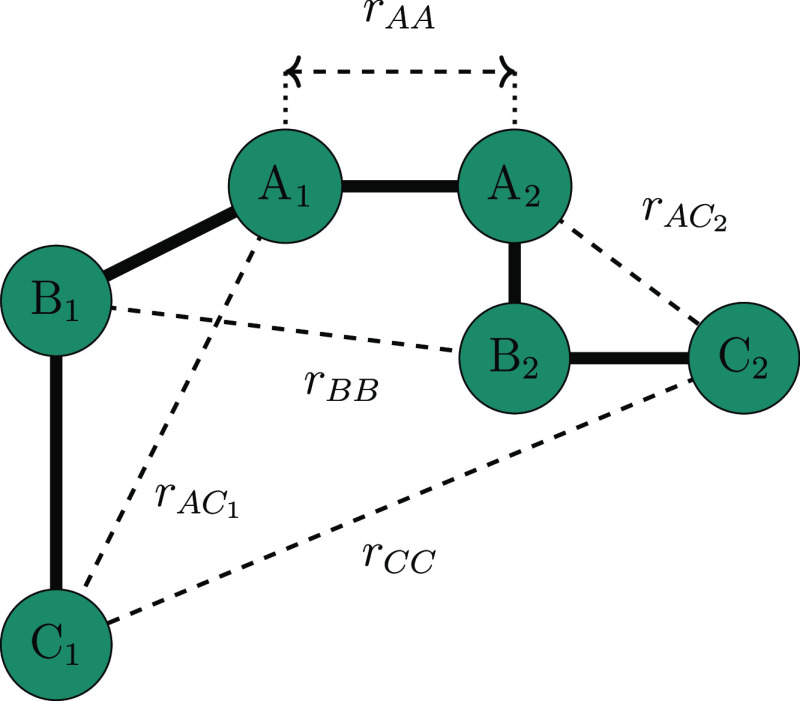
Schematic defining the different scalar inputs used as the input for the UMAP dimensionality reduction algorithm. Distances are calculated from the geometric center of the central five-membered rings of each fluorene monomer.

To determine the different typical conformations F8BT chains adopt within the amorphous phase, we used the uniform manifold approximation and projection (UMAP) dimensionality reduction algorithm to obtain a two-dimensional embedded space (with *n*_neighbors_ = 6).^72^ For UMAP, it is a requirement that the *min*_*dist* hyperparameter is set to 0.0 in order to subsequently cluster in the resultant embedding. We subsequently used HDBSCAN to perform the clustering in the UMAP embedding (*min*_*cluster*_*size* = 10; *cluster*_*selection*_*epsilon* = 0.85).^73^ The hyperparameters for UMAP and HDBSCAN detailed previously were selected iteratively for each system, and the physical meaningfulness of the resultant clusters was tested by comparing their respective physical characteristics. We use a relatively low-dimensional input space choosing descriptors informed by our previous experience with other polymers.^74^Figure 6 depicts the five input distances schematically, where each node represents a monomer in F8BT.

For each conformation *i*, the five distances described in Figure 6 make up elements of the input vector **r**_*i*_ = [*r*_*AA*_, *r*_*BB*_, *r*_*CC*_, max(*r*_*AC*_1__, *r*_*AC*_2__), min(*r*_*AC*_1__, *r*_*AC*_2__)]. Note that the ordering procedure for the final two elements is required to mitigate the symmetry of the molecule. Subsequently, each of these vectors become rows in a matrix used as the input for UMAP. Columns 1, 2, and 3 are normalized independently, while columns 4 and 5 are normalized together. Each normalization is performed such that the largest element in a given column (or columns 4 and 5 together) is set to 1.

#### Radial Distribution Functions

We use radial distribution functions (RDF) to understand the interactions of F8BT with its local environment, whether in the melt or solution phase. The RDF of two atomic species, *a* and *b*, is defined as
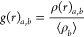
1where ρ(*r*)_*a*,*b*_ is the density of atom species *b* at distance *r* from atom species *a*, and ⟨ρ_*b*_⟩ is the average density of type *b* atoms in the simulation box. Where applicable, RDFs were processed using the Savitzky-Golay digital filter.^75^

#### Enrichment Indices

The enrichment (ϵ) of a given property (*p*) of a subset of molecular conformations (*c*) within the whole population of conformations (*C*) is defined as
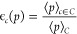
2In this work, we calculate the enrichment of ring stacking and intra- and intermolecular side chain contacts for the distinct conformational clusters identified in amorphous F8BT.

#### Structural Analysis of Polymer Chains

The extension length (*d*) of each polymer chain is defined as the scalar distance between the terminal FL carbon and terminal BT carbon of the polymer chain. The octyl side chain extension length is defined as the scalar distance between the terminal octyl methyl carbon and bridging carbon of the fluorene ring to which the octyl chain is attached.

#### Calculation of Contact Maps

Contact maps between the FL and BT rings that make up the F8BT backbone were calculated by identifying the specific atomistic intermolecular interactions between different rings. Interactions were assigned by identifying pairs of atoms found closer together than the cutoff distance, *r*_cut_ = 5 Å. Contact maps were then normalized by dividing each value of a given contact map by its maximum value, such that the contact map values range from 0 to 1, with a value of 1 denoting the most probable interaction.

### Theoretical Modeling of the Ring Stacking Network

A theoretical analysis of the amorphous polymer ring stacking network was performed using the polymer stacking degree distribution as the only input from the MD simulations. The approach is based on the cavity approach to percolation formulated originally by Karrer et al.,^76^ supplemented by techniques to expose the full heterogeneity in the problem and to then subsequently analyze it in the thermodynamic limit, as described by Kühn and Rogers.^77^ Full details of the calculations performed are provided in the Supporting Information (‘Theoretical Modeling of the Ring Stacking Network’).

## Supporting Information Available

F8BT chemical structure; intermonomer dihedral distributions; solvation mechanisms of F8BT in water and THF; quantifying polymer deformation and relaxation; reparameterization of F8BT classical force field; validation of the F8BT force field; theoretical modeling of ring stacking network (PDF)
