# Supplementary material for: Conformational Heterogeneity and Interchain Percolation Revealed in an Amorphous Conjugated Polymer
Source: ACS Nano. 2022 Sep 14;16(9):14432–42. doi: 10.1021/acsnano.2c04794 (PMC9527807; doi:10.1021/acsnano.2c04794)
Supplement: Supplementary file 1 — nn2c04794_si_001.pdf [file nn2c04794_si_001.pdf]

Supporting Information for "Conformational Heterogeneity and Interchain Percolation Revealed in an Amorphous Conjugated Polymer"

Robert M. Ziolek,[*,†] Alejandro Santana-Bonilla,[‡] Raquel López-Ríos de Castro,[¶,†] Reimer Kühn,[§] Mark Green,[‖] and Christian D. Lorenz[*,†]

†*Biological Physics and Soft Matter Group, Department of Physics, King's College London, London, WC2R 2LS, United Kingdom*

‡*Department of Physics, King's College London, London, WC2R 2LS, United Kingdom*

¶*Department of Chemistry, King's College London, London, SE1 1DB, United Kingdom*

§*Department of Mathematics, King's College London, London, WC2R 2LS, United Kingdom*

‖*Photonics and Nanotechnology Group, Department of Physics, King's College London, London, WC2R 2LS, United Kingdom*

E-mail: robert.ziolek@kcl.ac.uk; chris.lorenz@kcl.ac.uk

Chemical Structure of F8BT

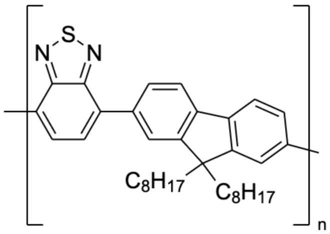

Figure S1: Chemical structure of F8BT. Hydrogen atoms are implied and in this work, $n = 6$.

Intermonomer Dihedral Distributions

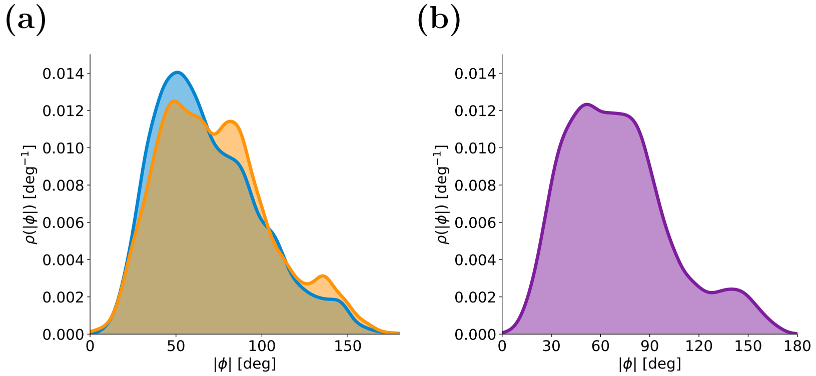

Figure S2: Intermonomer dihedral distributions. Intermonomer dihedral distributions for (a) water (blue) and THF (orange) and (b) amorphous F8BT at 350 K.

Solvation Mechanisms of F8BT in Water and THF

Figure S3 shows radial distribution functions ($g(r)$) and associated coordination numbers that describe the solvation mechanisms of F8BT in water (Figure S3(a)) and THF (Figure S3(b)). F8BT is insoluble in aqueous solution. In water, the benzothiadiazole ring is more strongly hydrated than the fluorene moiety. The highest level of solvation in the benzothiadiazole ring is seen for the S atom. Similarly, we observe that the C_D and C_E atoms are the most hydrated fluorene ring atoms, as these atoms are those least shielded by the octyl side chains. We observe preferential solvation of specific benzothiadiazole and fluorene atoms in water, while for F8BT in THF (Figure S3(b)), solvation is almost uniform across each of the benzothiadiazole and fluorene rings, i.e., the respective coordination numbers for benzothiadiazole and fluorene rings are almost the same. This indicates that both benzothiadiazole and fluorene ring atoms are not shielded from, and interact strongly with, THF (as opposed to water, in which we observe significantly lower levels of fluorene hydration than for benzothiadiazole).

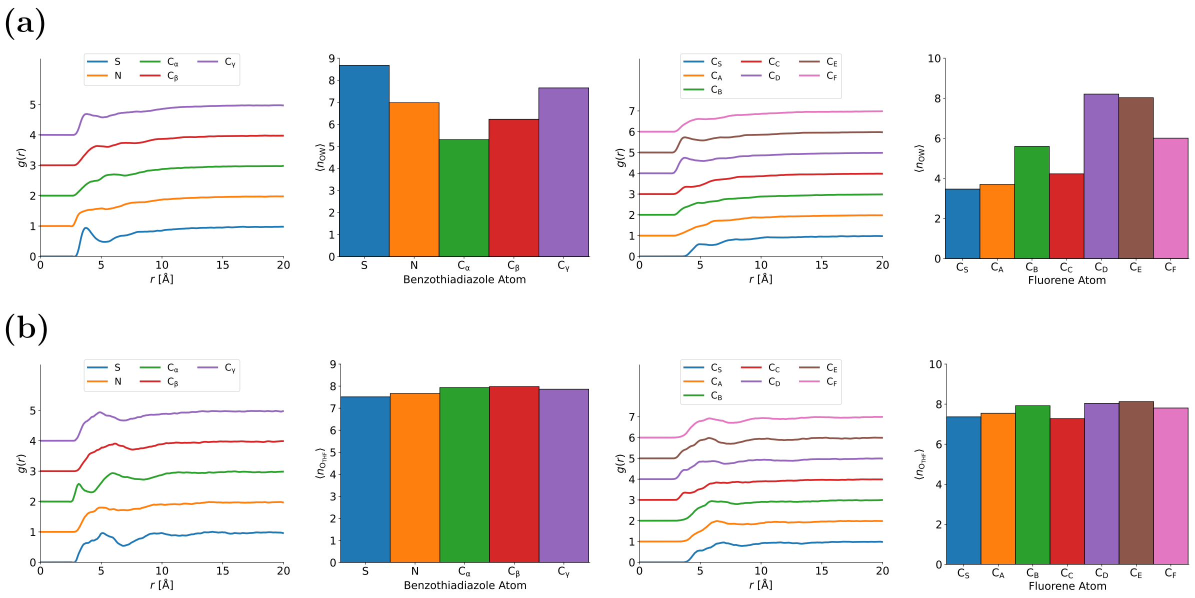

Figure S3: F8BT ring solvation in (a) water and (b) THF. A cutoff value of 7.5 Å was used to calculate the coordination numbers shown as bar charts. RDFs are vertically shifted by 1 for clarity.

Similar relative behaviour is also observed in the solvation mechanisms of the octyl side chains in water and THF (Figure S4). The coordination number of THF around the alkyl chain increases monotonically moving down the chain from the fluorene ring (with the exception of the terminal methyl group, which is expected to be due to the slight difference in steric hindrance conferred by the methyl H atoms). Conversely, for the case of F8BT in water, we note that preferential hydration is experienced by C3 compared to atoms we might expect to be found further into aqueous solution (C4, C5, and C6). This indicates that hydrophobic collapse of the side chains reduces solvent interactions with the side chains.

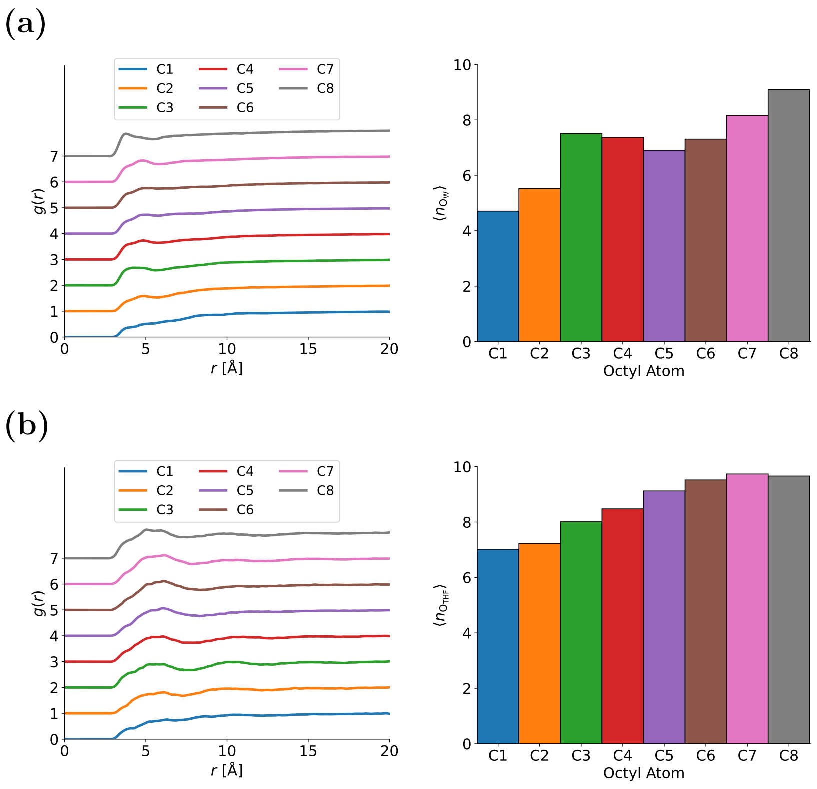

Figure S4: F8BT side chain solvation in (a) water and (b) THF. A cutoff value of 7.5 Å was used to calculate the coordination numbers shown as bar charts. RDFs are vertically shifted by 1 for clarity.

Quantifying Polymer Deformation and Relaxation

The following quantity provides a simple metric by which to compare the dynamics of polymer chains in different conditions (melt phase and in solution) and at different temperatures.[1] The root-mean squared deviation (RMSD) of a polymer chain given a lag time, τ, is defined as

$$\mathrm{RMSD}\left(\mathbf{r}(t), \mathbf{r}(t+\tau)\right) = \sqrt{\frac{1}{N}\sum_{i}^{N}(\mathbf{r}_i(t) - \mathbf{r}_i(t+\tau))^2} \tag{1}$$

Now we define $\Gamma(\tau) \equiv \langle \mathrm{RMSD}\left(\mathbf{r}(t), \mathbf{r}(t+\tau)\right)\rangle$, the RMSD as a function of the lag time, averaged over all molecules and different start times t. Assuming that $\Gamma \rightarrow \Gamma_{\mathrm{max}}$ as $\tau \rightarrow \infty$, we model the relaxation process using a simple first order ordinary differential equation (ODE):

$$\frac{\mathrm{d}\Gamma}{\mathrm{d}\tau} = k(\Gamma_{\mathrm{max}} - \Gamma) \tag{2}$$

Integrating the ODE and applying the initial conditions $\Gamma = 0$ when $\tau = 0$ yields

$$\Gamma_{\mathrm{max}} - \Gamma(\tau) = A\exp(-k\tau) \tag{3}$$

We evaluate $A = \Gamma_{\mathrm{max}}$ and as such, letting $k = \tau_{\mathrm{r}}^{-1}$ in addition, we obtain

$$\Gamma(\tau) = \Gamma_{\mathrm{max}}(1 - \exp(-\tau/\tau_{\mathrm{r}})) \tag{4}$$

We have defined $k = \tau_{\mathrm{r}}^{-1}$, where the constant τ_{r} is the relaxation time, a measure of the relaxation timescale of the chains according the evolution of $\Gamma(\tau)$. Therefore when $\tau = \tau_{\mathrm{r}}$, $\Gamma(t = \tau_{\mathrm{r}}) = \Gamma_{\mathrm{max}}(1 - e^{-1}) \approx 0.63\Gamma_{\mathrm{max}}$. The delay time measures the time by when the polymer has lost its memory of its initial conformation, acting as a timescale by which to measure chain relaxation dynamics. Furthermore, the value of Γ_{max} gives a sense of how free the polymer is to explore different conformations, i.e. it is a measure of deformability. We calculate Γ_{max} for $\tau = 1.5$ ns.

Reparametrization of the F8BT Force Field

Initial structures of 'F0BT' chains (i.e., F8BT chains with their octyl side chains replaced by H atoms) were obtained using the semi-empirical xTB-GFN2 method as implemented in the xTB package.[2] Note that the octyl chains present in F8BT were not included in these calculations since they have been shown to hinder convergence and accuracy of geometry optimisations.[3] Furthermore, they were not included in the F0BT chains used to calculate partial charges for similar reasons.

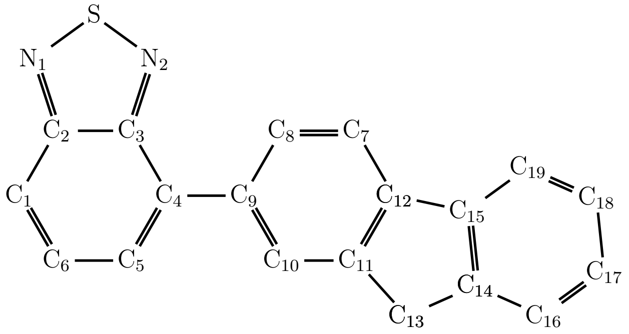

Figure S5: Chemical structure of the F0BT monomer. Note that hydrogen atoms are implicit.

Reparametrization of Partial Charges

F0BT oligomers were used to determine partial charges using the RESP method. Initial structures of F0BT oligomers were minimized using the xTB-GFN2 semi-empirical method before DFT energy minimization was performed. Full geometry optimisations were conducted on each F0BT chain. Using the outputted electron density, the Multiwfn package was used to calculate partial charges using the widely-used restrained electrostatic potential (RESP) method.[4] No constraints were applied when generating the partial charges, with respect to symmetry or with reference to the normal behaviour of the CHARMM force fields. Subsequently, the octyl alkyl chains were parameterised using the CHARMM General Force Field and merged with the calculated charges for the rest of the molecule. Each alkyl carbon atom that is connected to a F0BT carbon had its charge modified simply as

$q_{\text{C,new}} = q_{\text{C,orignal}} + q_{\text{H,removed}}$. Partial charges were then directly implemented in the GROMACS topology file, which is available as part of the Supporting Information.

An interesting comparison can be made between the CHARMM36 and RESP partial charges for the F0BT monomer. In donor-acceptor polymers such as F8BT, one can consider the donor unit (here fluorene) to undertake partial charge transfer towards the acceptor unit (here benzothiadiazole). This is reflected in the large, positive partial charge of C9 as calculated by RESP, which is not reflected in the CHARMM partial charge.

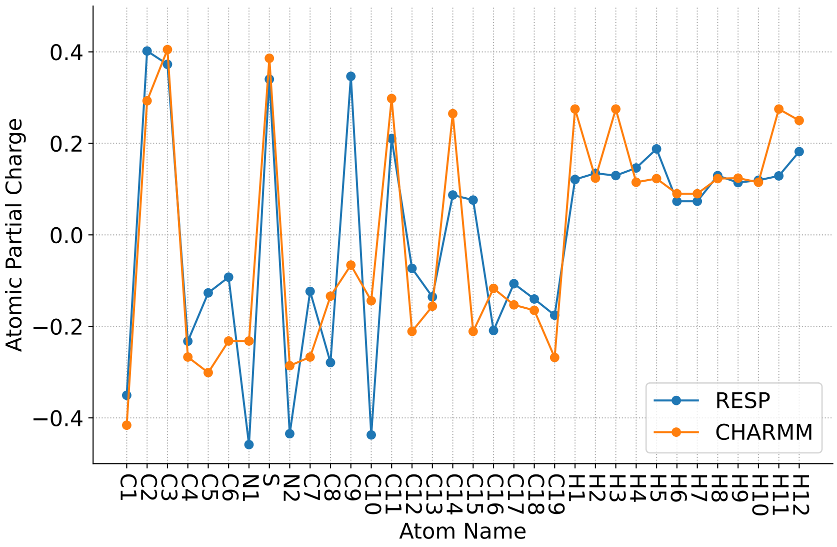

Figure S6: F0BT monomer partial charges from RESP and CHARMM. Atom names are the same as in Figure S5. Note that CHARMM underestimates the N partial charges and does not account for partial charge transfer from fluorene to benzothiadiazole, as indicated clearly by the respective C9 partial charges.

Reparametrization of Covalent Bond and Bond Angle Terms

We performed relaxed geometry scans to obtain energy profiles for covalent bond and bonded angle interactions that are poorly described by the classical force field, either to flagged by the large penalties arising in the initial parameterization, or because the chemical analogy made in the initial parameterisation was not appropriate. The geometry scans were performed on an F0BT monomer.

1. Calculate an approximate initial geometry using the semi-empirical GFN2-xTB method with the xTB package[2]

2. For each bonded interaction of interest (bond or angle), calculate the potential energy surface using relaxed scans by DFT calculations (B3LYP exchange-correlation functional[5,6] and def2-TZVPP Karlsruhe basis set) using the Orca quantum chemistry package[7]

3. Fit the target potential energy surfaces with an appropriate classical force field function (we used non-linear least squares as implemented in the scipy Python package)

Covalent bonds are described in the CHARMM force field as $V(r) = k_r(r - r_0)^2$, with k_r having units of $\mathrm{kcal\,mol^{-1}\,\AA^{-2}}$ and r_0 having units of Å, which we also used for the fitting procedure. However, direct implementation into the GROMACS topology file requires $V(r) = \frac{k_r}{2}(r - r_0)^2$, with k_r having units of $\mathrm{kJ\,mol^{-1}nm^{-2}}$ and r_0 having units of nm.

Table 1: Fitting results for select covalent bond interactions converted to CHARMM units. Note that the original CHARMM k_r values were used in our final force field.

Bond	k_r [$\mathrm{kcal\,mol^{-1}\,\AA^{-2}}$]	r_0 [Å]
C2-C3	457	1.45
C2-N1	437	1.35
N1-S	342	1.45

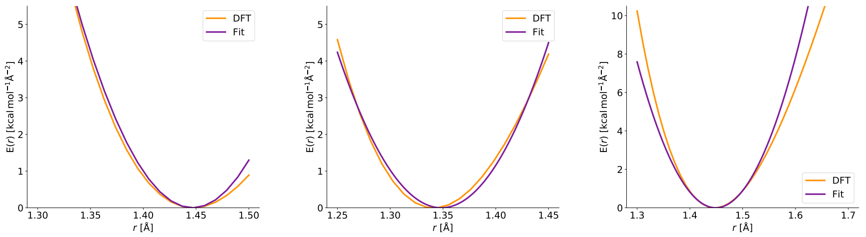

Figure S7: DFT bond energy scans and fitted force field functions for C2-C3, C2-N1, and N1-S (left to right).

Bonded angle deformations are described in the CHARMM force field as $V(\theta) = k_\theta(\theta - \theta_0)^2$, which we also used for the fitting procedure. However, for direct implementation into the

GROMACS topology file requires $V(\theta) = \frac{k_\theta}{2}(\theta - \theta_0)^2$, with k_r having units of $\mathrm{kJ\,mol^{-1}rad^{-2}}$ and r_0 having units of deg.

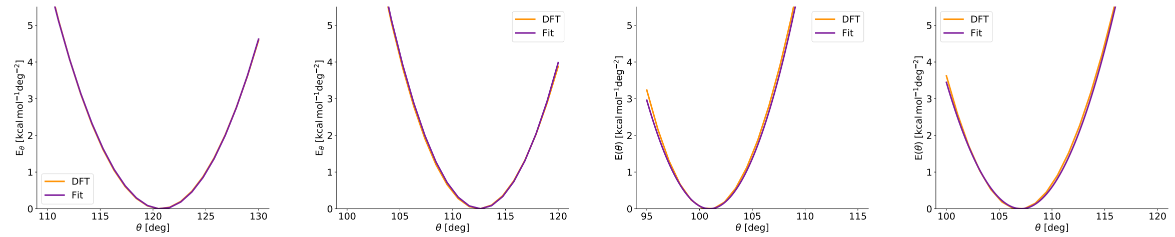

Figure S8: DFT angle deformation energy scans and fitted force field functions for C1-C2-C4, C1-C2-N2, N1-S-N2, and C2-N2-S (left to right).

Table 2: Fitting results for bonded angles in the F0BT monomer converted to CHARMM units. Note that the original CHARMM k_θ values were used in our final force field.

Angle	k_θ $[\mathrm{kcal\,mol^{-1}rad^{-2}}]$	θ_0 [deg]
C1-C2-N2	239	113
C1-C2-C4	178	121
C2-N2-S	226	107
N1-S-N2	275	101

Reparametrization of the Donor-Acceptor Dihedral Angle

We followed a similar approach to that of Wildman and co-workers to obtain suitable parameters to accurately describe the donor-acceptor dihedral angle.[3]

1. Build a representative oligomer and calculate an approximate initial geometry using the semi-empirical GFN2-xTB method with the xTB package[2]

2. For the dihedral of interest, calculate the potential energy surface using relaxed scans by DFT (B3LYP exchange-correlation functional[5,6] and def2-TZVPP Karlsruhe basis set) using the Orca quantum chemistry package[7]

3. Scan each of the bonded interactions with the Gromacs MD simulation engine (with dihedral potential energy term under investigation set to 0) to obtain the background

correction energy (mitigating double counting from the quantum chemistry data used by the fitting process[3])

4. Subtract the background correction energy from the fitted functional forms to obtain the final target energy function for the dihedral interaction

5. Fit the target potential energy surfaces with an appropriate classical force field function (we used non-linear least squares as implemented in the scipy Python package)

Figure S9 shows the 'push-pull' intermonomer dihedral pair used in the dihedral implementation in this work. The DFT and MD background energy scans were performed on the left-hand dihedral (note that this choice is arbitrary).

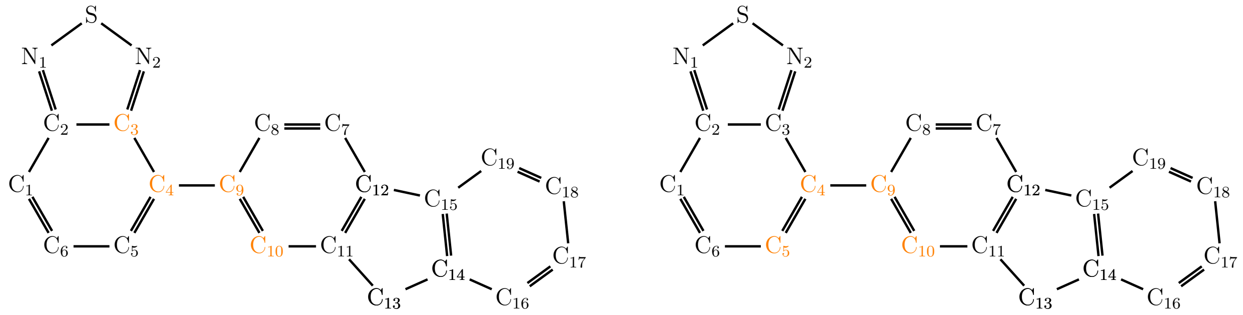

Figure S9: The 'push-pull' intermonomer dihedral pair.

Dihedral rotations are described using the following terms in the force field, with force constants and phase differences listed in Table 3.

$$V(\phi) = \sum_{n=1}^{5} k_{\phi,n}(1 + \cos(n\phi - \delta_n)) \tag{5}$$

Figure S10 shows the results from each section of the dihedral angle fitting procedure. Note the large contribution made by the background effects of the classical FF (gray line) and that the difference between the DFT dihedral energy (blue) and the overall classical FF dihedral energy as implemented in this work (green) is within chemical accuracy (i.e., a difference of less than 4 kJ mol^{-1}) for all dihedral angles.

Table 3: Fitting results for the donor-acceptor dihedral as implemented in the GROMACS topology file.

$\phi_{C_3C_4C_9C_{10}}$	n	$k_{\phi,n}$ [kJ mol^{-1}]	δ_n [deg]
	1	2.14	180
	2	−9.94	0
	3	0.58	0
	4	1.86	180
	5	0.33	180
$\phi_{C_5C_4C_9C_{10}}$	n	$k_{\phi,n}$ [kJ mol^{-1}]	δ_n [deg]
	1	2.14	0
	2	−9.94	180
	3	0.58	180
	4	1.86	0
	5	0.33	0

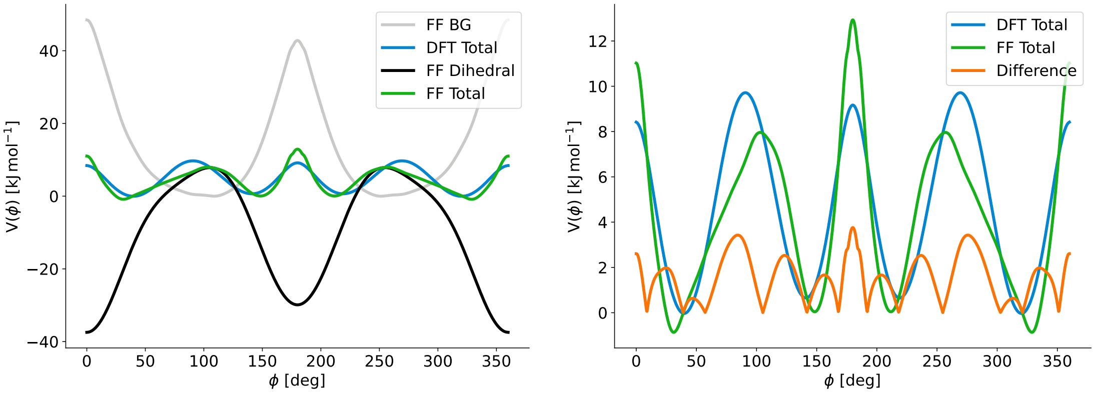

Figure S10: Final fitting results for the intermonomer dihedral angle potential.

Validation of the F8BT Force Field

While direct comparisons to the experimental structure of the amorphous state are not possible given a lack of experimental methods to probe the structure at the atomistic level of detail, experiments do however provide a suitable point of reference by which to test the validity of the new force field. F8BT is insoluble in water but highly soluble in tetrahydrofuran (THF).[8] To test the solubility of F8BT as modelled by our new force field we performed two simulations, both containing 17 F8BT chains in water and THF respectively.

Simulation Details. F8BT was modeled using the reparameterized CHARMM-based force field[9,10] developed as part of this work. Before use in any production simulation, the F8BT chain was subject to high temperature dynamics *in vacuo* to randomize its inter-monomer dihedral distribution. We note that frequent transitions between different dihedral states are observed in all simulations in addition. Water was treated with the CHARMM-modified TIP3P model[11] and tetrahydrofuran (THF) was modeled using the CHARMM36 force field.[9,10] The MD simulations were performed using the GROMACS 2019 simulation engine.[12,13] In all simulations, the Lennard-Jones and Coulomb interaction cut-off distances were set to 12 Å. The particle-mesh Ewald method was used to calculate long-range electrostatic interactions. Periodic boundary conditions were applied in all dimensions in all simulations. A 1 fs timestep was used for all equilibration and production simulations. The leapfrog integration scheme was used in both simulations. For both water and THF simulations, 17 F8BT chains were randomly placed in a simulation box measuring $110 \times 110 \times 110$ Å^3. Solvent molecules were added to yield a system containing F8BT with a concentration of 10.0 wt. % (see Table 4 for details). Both systems were once more subjected to energy minimization by steepest descent before the temperature was equilibrated to 350 K for 100 ps using the Nosé-Hoover thermostat. Subsequently, the production simulation for the F8BT chain in water was performed using the velocity-rescale thermostat[14] (target temperature of 353 K) and the Parrinello-Rahman barostat[15] (target pressure of 1 atm) for 200 ns to allow for any molecular aggregation to be observed.

Table 4: Details of the two simulated systems.

System	n_{atoms}	n_{F8BT}	n_{solvent}	Final box size [Å^3]	Conc. [wt. %]
F8BT chains in H_2O	97 213	17	29 673	100.6×100.6×100.6	10.0
F8BT chains in THF	104 485	17	7 407	109.8×109.8×109.8	10.0

Analysis Methods. Simulation analysis was performed using in-house Python scripts, which make wide use of the MDAnalysis[16] and NetworkX packages.[17] Simulation visualizations were produced using VMD.[18] Clustering was performed using a graph-theoretic technique described fully elsewhere,[19] using a cutoff distance of 20 Å was used between the methylene carbon atom of the central fluorene moiety of each polymer. The largest aggregate time series was then calculated, as well as the aggregate size distribution at stationarity. A burn-in time of 150 ns was selected by considering the time series results in Figure S11(c).

Summary of Simulations. We find that our force field correctly describes the solubility of F8BT in water and THF. Figure S11(a) shows that after 200 ns of simulation time, all of the F8BT chains phase separate to form a single aggregate, which traverses the periodic boundaries of the simulation box. Whereas after the same amount of time, the F8BT chains in THF are observed to remain well-dispersed (Figure S11(b)). These observations are quantified by calculating the fraction of F8BT chains found in the largest aggregate for each solvent, which is presented as the time series in Figure S11(c), where we see that after a burn-in time of 150 ns, at stationarity, essentially all of the F8BT chains are found in one aggregate in water, while no large clusters are formed in THF. The probability distributions of aggregate size at stationarity are shown for water and THF in Figures S11(d,e) respectively, which highlight the qualitatively different behavior observed for the two solvents in the simulations, in agreement with the experimental solubility of F8BT in both water and THF.

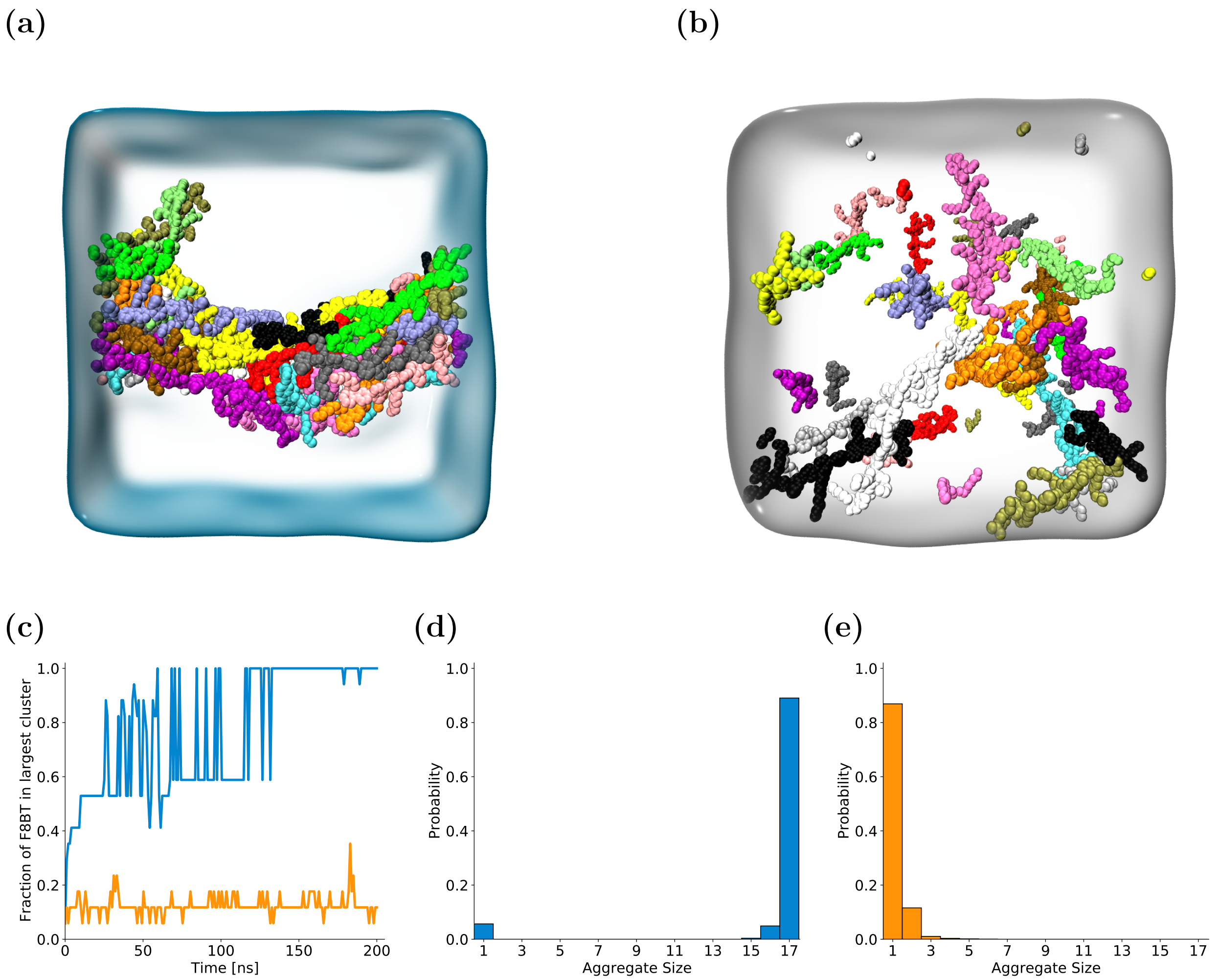

Figure S11: Snapshots of the final frame of each simulation showing (a) the aggregation of F8BT chains in water and (b) the dispersion of individual F8BT chains in THF. (c) Fraction of F8BT molecules in the largest aggregate over time for both systems (water in blue, THF in orange). Probability distributions of F8BT aggregate size for chains in (d) water and (e) THF. N.B.: a burn-in time of 150 ns was used to calculate these distributions at stationarity with respect to aggregation in both systems.

Theoretical Modelling of the Ring Stacking Network

The following is a summary of key concepts that go into the analysis of inter-chain percolation in a glassy configuration of a system of conjugated polymers. The analysis conceives of the amorphous polymer mixture as a network of randomly interlinked polymers, with the links realized through ring stacking interactions as described in the main paper. We will in the following, therefore, refer to polymers also as nodes or vertices of a network or graph, and to the ring stacking interactions as the edges. The theory described below is based on the cavity-approach to percolation formulated by Karrer et al.,[20] supplemented by techniques to expose the full heterogeneity in the problem *and* to analyse it in the thermodynamic limit as described in Kühn and Rogers.[21]

Cavity Approach to Percolation

As input to our analysis, we use the empirically determined probability mass function (pmf) of the polymer stacking degree determined from the simulation, as displayed in Figure 3(b) in the main text. We will assume that the configuration is a random realization of an amorphous configuration of a network giving rise to the pmf of polymer stacking degrees, and that this stacking degree distribution is characteristic of the true limiting stacking degree pmf one would observe in the thermodynamic limit $N \to \infty$ of infinite system size.

Probabilistic Description

Our analysis is based on a probabilistic analysis of bond percolation in large heterogeneous networks, devised in Karrer et al.[20] They consider a network or graph $G = (V, E)$, with V denoting the set of vertices or nodes of the graph, and E the set of edges or links, the polymer stacking interactions in the case at hand. We use $N = |V|$ to denote the size of the graph and $i \in \{1, \ldots, N\}$ to label the nodes.

Bond percolation is a process by which each edge $(ij) \in E$ is either kept with probability

p or deleted with probability $1-p$. A probabilistic description of percolation is obtained by noting that the configuration of connected clusters will be random and be determined by the random realization of the percolation process. For the purpose of the present study, we will eventually be interested in the results of the probabilistic description in the limit $p \to 1$, where all edges of the system are kept.

In Karrer et al., the authors analyse the probabilities $\pi_i(s)$, $i \in \{1, \dots, N\}$, of individual nodes i to belong to a cluster of finite size s, $s \in \mathbb{N} = \{1, 2, \dots\}$.[20] Note that, in the thermodynamic limit $N \to \infty$, the π_i are *not* necessarily normalized probability mass functions, as a fraction of nodes may sit in a percolating or giant connected cluster (GCC) of the system which is not of *any* finite size. The $\pi_i(s)$ satisfy the following equation[20]

$$\pi_i(s) = \sum_{\boldsymbol{s}_{\partial i}} \Big[\prod_{j \in \partial i} \pi_j^{(i)}(s_j) \Big] \delta_{s, 1+\sum_{j \in \partial i} s_j} \tag{6}$$

in which $\boldsymbol{s}_{\partial i} = \big(s_j\big)_{j \in \partial i}$ denotes the collection of cluster sizes s_j that can be reached through the edges (ij) connected to i, and $\pi_j^{(i)}(s_j)$ denotes the probability that s_j nodes can be reached from i trough the edge (ij). This equation expresses the logic that the size of the cluster containing i must be equal to the sum of the sizes of the clusters that can be reached through edges connecting to i, plus 1 (accounting for the node i itself).

For the $\pi_j^{(i)}(s_j)$ a set of self-consistency equations is derived,[20] which express the same line of reasoning, viz.

$$\pi_j^{(i)}(s_j) = (1-p)\delta_{s_j,0} + p \sum_{\boldsymbol{s}_{\partial j \setminus i}} \Big[\prod_{\ell \in \partial j \setminus i} \pi_\ell^{(j)}(s_\ell) \Big] \delta_{s_j, 1+\sum_{\ell \in \partial j \setminus i} s_\ell} \ , \tag{7}$$

in which the first contribution corresponds to the configuration that the edge (ij) is absent, while the second contribution describes the configuration in which the edge (ij) is present.

These equations are exact only on trees. However they provide excellent approximations for large finitely connected systems, as the one under study, and are known to become

asymptotically exact in the thermodynamic limit.[20–22]

Equations (6) and (7) are most efficiently analysed using probability generating functions or z-transforms corresponding to $\pi_i(s)$ and $\pi_j^{(i)}(s)$, respectively, defined as[20]

$$G_i(z) = \sum_{s\geq 1} \pi_i(s) z^s \tag{8}$$

and

$$H_j^{(i)}(z) = \sum_{s\geq 0} \pi_j^{(i)}(s) z^s \ . \tag{9}$$

For these, Eqs. (6) and (7) entail

$$G_i(z) = z \prod_{j\in\partial i} H_j^{(i)}(z) \tag{10}$$

$$H_j^{(i)}(z) = 1 - p + pz \prod_{\ell\in\partial j\setminus i} H_\ell^{(j)}(z) \ , \tag{11}$$

so these equations can be solved independently for each z.

Noting that $G_i(1)$ is the probability that site i belongs to a cluster of *any* finite size s, one can conclude that

$$g_i = 1 - G_i(1) = 1 - \prod_{j\in\partial i} H_j^{(i)}(1) \tag{12}$$

is the probability that site i belongs to the giant cluster, and thus

$$\bar{g} = \frac{1}{N}\sum_{i=1}^{N} g_i \tag{13}$$

gives the average fraction of sites occupied by the giant cluster.

Given a site i is not on the giant cluster, the expected size of the finite cluster to which i belongs is

$$\langle s_i \rangle = \frac{\sum_s s\pi_i(s)}{\sum_s \pi_i(s)} = \frac{G_i'(1)}{G_i(1)} = 1 + \sum_{j\in\partial i} \frac{H_j^{(i)\prime}(1)}{H_j^{(i)}(1)} \ . \tag{14}$$

Its evaluation requires the z-derivative $H_j^{(i)\prime}(z)$ evaluated at $z = 1$, which is obtained from

(11) as

$$H_j^{(i)\prime}(1) = p\left[1 + \sum_{\ell\in\partial j\setminus i} \frac{H_\ell^{(j)\prime}(1)}{H_\ell^{(j)}(1)}\right] \prod_{\ell\in\partial j\setminus i} H_\ell^{(j)}(1) \ . \tag{15}$$

Equations (11) (at $z = 1$) and (15) can efficiently be solved by forward iteration using random initial conditions, and the solutions can then be used to evaluate (12) and thereby the average fraction $\bar{g}$ of sites contained in the giant cluster (13) as well as the average sizes $\langle s_i \rangle$ of finite clusters (14) containing individual nodes in the system. Averages are here to be understood as averages over many instances of the percolation problem on the same large complex network, so using the present setup avoids simulating many instances of a percolation experiment for the purpose of averaging. Given a large single instance of a complex network, the solution of (11) will in general be heterogeneous across bonds (ij) entailing that the g_i as well as the $\langle s_i \rangle$ will be heterogeneous across nodes. Note, however, that the analysis described above requires complete knowledge of the original graph $G = (V, E)$. In the absence of such knowledge, one can resort to an analysis of the system in the thermodynamic limit $N \to \infty$ that only requires knowledge of the limiting degree distribution $\boldsymbol{p} = (p_k)_{k\geq 0}$ of the nodes in the system.

The Large System Limit

Assuming that, in the limit of infinite system size, there is a limiting joint probability density function of the $H_j^{(i)} \equiv H_j^{(i)}(1)$ and the $H_j^{(i)\prime} \equiv H_j^{(i)\prime}(1)$, such that

$$\mathrm{d}\tilde{\pi}(h, h') \equiv \tilde{\pi}(h, h')\, \mathrm{d}h\mathrm{d}h' = \mathrm{Prob}\Big(H_j^{(i)} \in (h, h + \mathrm{d}h], H_j^{(i)\prime} \in (h', h' + \mathrm{d}h']\Big) \ , \tag{16}$$

one can use stochastic self-consistency arguments in Eqs. (11) and (15), as explained by Kühn and Rogers,[21] to obtain the following non-linear integral equation for this joint probability

density function

$$\tilde{\pi}(h,h') = \sum_{k\geq 1} \frac{k}{c} p_k \int \Big[\prod_{\ell=1}^{k-1} \mathrm{d}\tilde{\pi}(h_\ell,h'_\ell)\Big] \delta\Big(h - \Big(1-p+p\prod_{\ell=1}^{k-1} h_\ell\Big)\Big) \times \delta\Big(h' - p\Big(1+\sum_{\ell=1}^{k-1}\frac{h'_\ell}{h_\ell}\Big)\prod_{\ell=1}^{k-1} h_\ell\Big). \quad (17)$$

Here $\frac{k}{c}p_k$ with $c = \langle k\rangle$ is the probability that a randomly chosen neighbour of a node has degree k, assuming as we do that there are no degree correlations in the system.

From its solution, which can be very efficiently obtained using a stochastic so-called population dynamics algorithm,[23] one obtains, with reference to Eq. (12), the probability density function $P(g)$ of the g_i[21] as

$$P(g) = \sum_k p_k \int \Big[\prod_{\ell=1}^{k} \mathrm{d}\tilde{\pi}(h_\ell,h'_\ell)\Big] \delta\Big(g - 1 + \prod_{\ell=1}^{k} h_\ell\Big) \quad (18)$$

and, with reference to Eq. (14), the probability density function $Q(s)$ of the $\langle s_i\rangle$ as

$$Q(s) = \sum_k p_k \int \Big[\prod_{\ell=1}^{k} \mathrm{d}\tilde{\pi}(h_\ell,h'_\ell)\Big] \delta\Big(s - 1 - \sum_{\ell=1}^{k}\frac{h'_\ell}{h_\ell}\Big) . \quad (19)$$

Note that the probability density function $Q(s)$ of the average sizes of clusters containing a randomly selected node is related to the cluster size distribution $P(s)$ via

$$Q(s) = \frac{s}{\langle s\rangle} P(s) , \qquad \text{with} \quad \langle s\rangle = \int \mathrm{d}s\, s\, P(s) , \quad (20)$$

which follows from the fact that each cluster of size s has s nodes which belong to it.

For the purposes of the present investigation we are interested in the $p \to 1$ limit of these identities and results. In this limit, the solution of the system Eqs. (11) of self-consistency equations can be shown to take values $H_j^{(i)} \equiv H_j^{(i)}(1) \in \{0,1\}$ and the solutions $H_j^{(i)}$ take the meaning of indicator variables, which take the value $H_j^{(i)} = 1$, if the edge (ij) does *not*

connect node i to the GCC, and the value $H_j^{(i)} = 0$, if it does. As a result, the g_i defined by Eq. (12), will in this limit themselves be indicator variables, taking the value $g_i = 1$ if i is part of the GCC, and $g_i = 0$, if it is not, with Eq. (12) in this limit expressing the logic that for a node to belong to the GCC, it must be connected to the GCC through at least one of its neighbours. In this sense the system Eqs. (11) of self-consistency equations at $z = 1$ provide a local algorithm that is able to reveal whether a node belongs to the GCC of a given network or not. One can similarly convince oneself that in the limit $p \to 1$ the solutions $H_j^{(i)\prime} \equiv H_j^{(i)\prime}(1)$ of the system (15) and (11) (for $z = 1$) of equations take values on the non-negative integers $\mathbb{N}_0 = \{0, 1, 2, \dots\}$ entailing that both $P(s)$ and $Q(s)$ will be supported on the positive integers. Results for $P(s)$ obtained from the large system limit of the theory as described above are displayed in Figure 3(c) in the main text. The only input that goes into obtaining them is the empirical degree distribution $\boldsymbol{p} = (p_k)$ of the polymer stacking interaction obtained from the MD simulations of amorphous F8BT which is displayed in Figure 3(b) in the main text. Results from theory and the numerical experiments are in excellent agreement, despite the moderate system size. This is in line with many other studies of percolation on complex networks, according to which finite-size corrections are expected to be small except in the immediate vicinity of the percolation transition. With the GCC occupying approximately 89% of the system, the present conditions do indeed place the simulated glassy polymer configuration far away from the percolation transition. The probability distribution of small clusters, as calculated by the methodology outlined here, is shown in Figure S12 below.

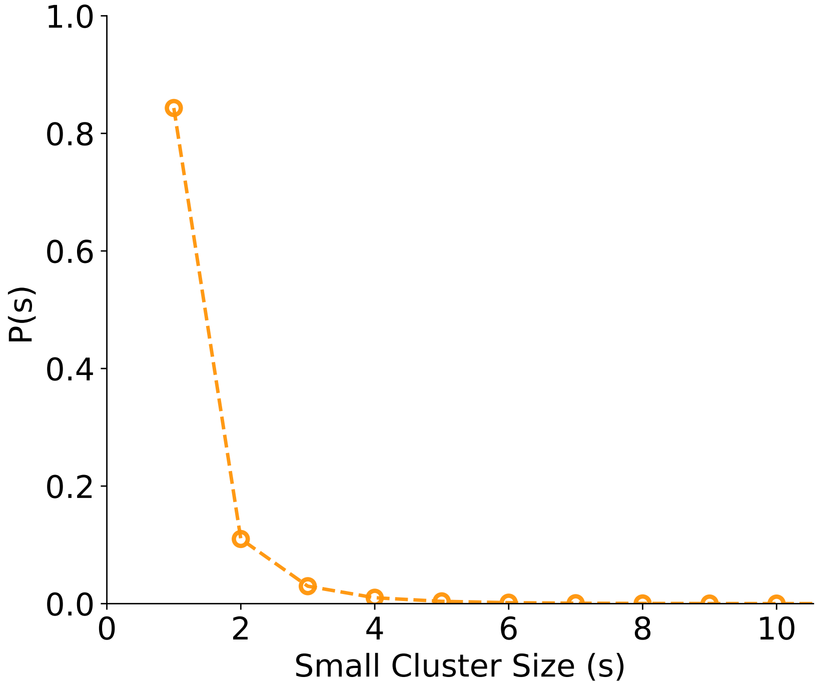

Figure S12: Probability distribution of small clusters.

References

(1) Wang, J.; Ferguson, A. L. A Study of the Morphology, Dynamics, and Folding Pathways of Ring Polymers with Supramolecular Topological Constraints Using Molecular Simulation and Nonlinear Manifold Learning. *Macromolecules* **2018**, *51*, 598–616.

(2) Bannwarth, C.; Ehlert, S.; Grimme, S. GFN2-xTB—An Accurate and Broadly Parametrized Self-Consistent Tight-Binding Quantum Chemical Method with Multipole Electrostatics and Density-Dependent Dispersion Contributions. *J. Chem. Theory Comput.* **2019**, *15*, 1652–1671.

(3) Wildman, J.; Repiscak, P.; Paterson, M. J.; Galbraith, I. General Force-Field Parametrization Scheme for Molecular Dynamics Simulations of Conjugated Materials in Solution. *J. Chem. Theory Comput.* **2016**, *12*, 3813–3824.

(4) Lu, T.; Chen, F. Multiwfn: A multifunctional wavefunction analyzer. *J. Comput. Chem.* **2012**, *33*, 580–592.

(5) Becke, A. D. A new mixing of Hartree–Fock and local density-functional theories. *J. Chem. Phys.* **1993**, *98*, 1372–1377.

(6) Lee, C.; Yang, W.; Parr, R. G. Development of the Colle-Salvetti correlation-energy formula into a functional of the electron density. *Phys. Rev. B* **1988**, *37*, 785–789.

(7) Neese, F. Software update: the ORCA program system, version 4.0. *Wiley Interdiscip. Rev. Comput. Mol. Sci.* **2018**, *8*, e1327.

(8) Urbano, L.; Clifton, L.; Ku, H. K.; Kendall-Troughton, H.; Vandera, K.-K. A.; Matarese, B. F. E.; Abelha, T.; Li, P.; Desai, T.; Dreiss, C. A.; Barker, R. D.; Green, M. A.; Dailey, L. A.; Harvey, R. D. Influence of the Surfactant Structure on Photoluminescent -Conjugated Polymer Nanoparticles: Interfacial Properties and Protein Binding. *Langmuir* **2018**, *34*, 6125–6137.

(9) Mackerell Jr., A. D.; Feig, M.; Brooks, C. L. Extending the Treatment of Backbone Energetics in Protein Force Fields: Limitations of Gas-Phase Quantum Mechanics in Reproducing Protein Conformational Distributions in Molecular Dynamics Simulations. *J. Comput. Chem.* **2004**, *25*, 1400–1415.

(10) Vanommeslaeghe, K.; Hatcher, E.; Acharya, C.; Kundu, S.; Zhong, S.; Shim, J.; Darian, E.; Guvench, O.; Lopes, P.; Vorobyov, I.; Mackerell Jr., A. D. CHARMM General Force Field: A Force Field for Drug-like Molecules Compatible with the CHARMM All-Atom Additive Biological Force Fields. *J. Comput. Chem.* **2010**, *31*, 671–690.

(11) Jorgensen, W. L.; Chandrasekhar, J.; Madura, J. D.; Impey, R. W.; Klein, M. L. Comparison of Simple Potential Functions for Simulating Liquid Water. *J. Chem. Phys.* **1983**, *79*, 926–935.

(12) Lindahl, E.; Hess, B.; van der Spoel, D. GROMACS 3.0: a package for molecular simulation and trajectory analysis. *J. Mol. Model.* **2001**, *7*, 306–317.

(13) Abraham, M. J.; Murtola, T.; Schulz, R.; Páll, S.; Smith, J. C.; Hess, B.; Lindahl, E. GROMACS: High performance molecular simulations through multi-level parallelism from laptops to supercomputers. *SoftwareX* **2015**, *1-2*, 19–25.

(14) Bussi, G.; Donadio, D.; Parrinello, M. Canonical sampling through velocity rescaling. *J. Chem. Phys.* **2007**, *126*, 014101.

(15) Parrinello, M.; Rahman, A. Polymorphic transitions in single crystals: A new molecular dynamics method. *J. Appl. Phys.* **1981**, *52*, 7182–7190.

(16) Michaud-Agrawal, N.; Denning, E. J.; Woolf, T. B.; Beckstein, O. MDAnalysis: A Toolkit for the Analysis of Molecular Dynamics Simulations. *J. Comput. Chem.* **2011**, *32*, 2319–2327.

(17) Hagberg, A. A.; Schult, D. A.; Swart, P. J. Exploring Network Structure, Dynamics, and Function using NetworkX. Proceedings of the 7th Python in Science Conference. Pasadena, CA USA, 2008; pp 11 – 15.

(18) Humphrey, W.; Dalke, A.; Schulten, K. VMD: Visual Molecular Dynamics. *J. Mol. Graph.* **1996**, *14*, 33–38.

(19) Ziolek, R. M.; Omar, J.; Hu, W.; Porcar, L.; González-Gaitano, G.; Dreiss, C. A.; Lorenz, C. D. Understanding the pH-Directed Self-Assembly of a Four-Arm Block Copolymer. *Macromolecules* **2020**, *53*, 11065–11076.

(20) Karrer, B.; Newman, M. E. J.; Zdeborová, L. Percolation on Sparse Networks. *Phys. Rev. Lett.* **2014**, *113*, 208702.

(21) Kühn, R.; Rogers, T. Heterogeneous Micro-Structure of Percolation in Sparse Networks. *Europhys. Lett.* **2017**, *118*, 68003.

(22) Newman, M. E. J. *Networks: an Introduction, 2nd Ed.*; Oxford Univ. Press: Oxford, 2018.

(23) Mézard, M.; Parisi, G. The Bethe Lattice Spin Glass Revisited. *Eur. Phys. J. B* **2001**, *20*, 217–233.
